# Learning mean-field equations from particle data using WSINDy

**DOI:** 10.1016/j.physd.2022.133406

**Published:** 2022-06-18

**Authors:** Daniel A. Messenger, David M. Bortz

**Affiliations:** Department of Applied Mathematics, University of Colorado Boulder, 11 Engineering Dr, Boulder, CO 80309, USA

**Keywords:** Data-driven modeling, Interacting particle systems, Weak form, Mean-field limit, Sparse regression

## Abstract

We develop a weak-form sparse identification method for interacting particle systems (IPS) with the primary goals of reducing computational complexity for large particle number N and offering robustness to either intrinsic or extrinsic noise. In particular, we use concepts from mean-field theory of IPS in combination with the weak-form sparse identification of nonlinear dynamics algorithm (WSINDy) to provide a fast and reliable system identification scheme for recovering the governing stochastic differential equations for an IPS when the number of particles per experiment N is on the order of several thousands and the number of experiments M is less than 100. This is in contrast to existing work showing that system identification for N less than 100 and M on the order of several thousand is feasible using strong-form methods. We prove that under some standard regularity assumptions the scheme converges with rate $O(N^{-1∕2})$ in the ordinary least squares setting and we demonstrate the convergence rate numerically on several systems in one and two spatial dimensions. Our examples include a canonical problem from homogenization theory (as a first step towards learning coarse-grained models), the dynamics of an attractive–repulsive swarm, and the IPS description of the parabolic–elliptic Keller–Segel model for chemotaxis. Code is available at https://github.com/MathBioCU/WSINDy_IPS.

## 1. Problem statement

Recently there has been considerable interest in the methodology of data-driven discovery for governing equations. Building on the Sparse Identification of Nonlinear Dynamics (SINDy) [1], we developed a weak form version (WSINDy) for ODEs [2] and for PDEs [3]. In this work, we develop a formulation for discovering governing stochastic differential equations (SDEs) for interacting particle systems (IPS). To promote clarity and for reference later in the article, we first state the problem of interest. Subsequently, we will provide a discussion of background concepts and current results in the literature.

Consider a particle system $X_{t}=(X_{t}^{\left(1\right)},\ldots ,X_{t}^{\left(N\right)})\in R^{Nd}$ where on some fixed time window t∈[0,T], each particle $X_{t}^{\left(i\right)}\in R^{d}$ evolves according to the overdamped dynamics

(1.1) $$dX_{t}^{\left(i\right)}=\left(-\nabla K*\mu_{t}^{N}\left(X_{t}^{\left(i\right)}\right)-\nabla V\left(X_{t}^{\left(i\right)}\right)\right)dt+\sigma (X_{t}^{\left(i\right)})dB_{t}^{\left(i\right)}$$

with initial data $X_{0}^{\left(i\right)}$ each drawn independently from some probability measure $\mu_{0}\in P_{p}(R^{d})$, where $P_{p}(R^{d})$ is the space probability measures on $R^{d}$ with finite pth moment.^1^ Here, K is the *interaction potential* defining the pairwise forces between particles, V is the *local potential* containing all exogenous forces, σ is the diffusivity, and ${\left(B_{t}^{\left(i\right)}\right)}_{i=1,\ldots ,N}$ are independent Brownian motions each adapted to the same filtered probability space (Ω, B, P, ($(F_{t}{)}_{t\geq 0}$). The *empirical measure* is defined

$$\mu_{t}^{N}≔\frac{1}{N}\sum_{i=1}^{N}\delta_{X_{t}^{\left(i\right)}},$$

and the convolution $\nabla K*\mu_{t}^{N}$ is defined

$$\nabla K*\mu_{t}^{N}(x)=\nabla \int_{R^{d}}K(x-y)\;d\mu_{t}^{N}(y)=\frac{1}{N}\sum_{i=1}^{N}\nabla K\left(x-X_{t}^{\left(i\right)}\right)$$

where we set ∇K(0)=0 whenever ∇K(0) is undefined. The recovery problem we wish to solve is the following.

(P) Let $X=(X_{t}^{\left(1\right)},\ldots ,X_{t}^{\left(M\right)})$ be discrete-time data at L timepoints $t\;≔(t_{1},\ldots ,t_{L}$ for M i.i.d. trials of the process (1.1) with $K=K^{⋆}$, $V=V^{⋆}$, and $\sigma =\sigma^{⋆}$ and let Y=X+ε be a corrupted dataset. For some fixed compact domain $D\subset R^{d}$ containing supp (Y), and finite-dimensional hypothesis spaces^2^ $H_{K}\subset L^{2}(D-D)$, $H_{V}\subset L^{2}(D)$, and $H_{\sigma }\subset L^{2}(D)$, solve

$$(\hat{K},\hat{V},\hat{\sigma })=\underset{K\in H_{K},V\in H_{V},\sigma \in H_{\sigma }}{\text{argmin}}{\left\|\nabla K-\nabla K^{⋆}\right\|}_{L^{2}(D-D)}\;\;+{\left\|\nabla V-\nabla V^{⋆}\right\|}_{L^{2}(D)}+\|\sigma -\sigma^{⋆}{\|}_{L^{2}(D)}.$$

The problem (P) is clearly intractable because we do not have access to $K^{⋆}$, $V^{⋆}$, or $\sigma^{⋆}$, and moreover the interactions between these terms render simultaneous identification of them ill-posed. We consider two cases: (i) ε≠0 and $\sigma^{⋆}=0$, corresponding to purely *extrinsic noise*, and (ii) ε=0 and $\sigma^{⋆}\neq 0$, corresponding to purely *intrinsic noise*. The extrinsic noise case is important for many applications, such as cell tracking, where uncertainty is present in the position measurements. In this case we examine ε representing i.i.d. Gaussian noise with mean zero and variance^3^ $\epsilon^{2}I_{d}$ added to each particle position in X. In the case of purely intrinsic noise, identification of the diffusivity $\sigma^{⋆}$ is required as well as the deterministic forces on each particle as defined by $K^{⋆}$ and $V^{⋆}$. A natural next step is to consider the case with both extrinsic and intrinsic noise. However, the combined noise case is sufficiently nuanced as to render it beyond the scope of the article, and we leave it for future work.

## 2. Background

Interacting particle systems (IPS) such as (1.1) are used to describe physical and artificial phenomena in a range of fields including astrophysics [4,5], molecular dynamics [6], cellular biology [7-9], and opinion dynamics [10]. In many cases the number of particles N is large, with cell migration experiments often tracking 10^3^ – 10^6^ cells and simulations in physics (molecular dynamics, particle-in-cell, etc.) requiring N in the range 10^6^ – 10^12^. Inference of such systems from particle data thus requires efficient means of computing pairwise forces from $O(N^{2})$ interactions at each timestep for multiple candidate interaction potentials K. Frequently, so-called *mean-field* equations at the continuum level are sufficient to describe the evolution of the system, however in many cases (e.g. chemotaxis in biology [11]) only phenomenological mean-field equations are available. Moreover, it is often unclear how many particles N are needed for a mean-field description to suffice. Many disciplines are now developing machine learning techniques to extract coarse-grained dynamics from high-fidelity simulations (see [12] for a recent review in molecular dynamics). In this work we provide a means for inferring governing mean-field equations from particle data assumed to follow the dynamics (1.1) that is highly efficient for large N, and is effective in learning mean-field equations when N is in range 10^3^ – 10^5^.

Inference of the drift and diffusion terms for stochastic differential equations (SDEs) is by now a mature field, with the primary method being maximum-likelihood estimation, which uses Girsanov’s theorem together with the Radon–Nikodym derivative to arrive at a log-likelihood function for regression. See [13, 14] for some early works and [15] for a textbook on this approach. More recently, sparse regression approaches using the Kramers–Moyal expansion have been developed [16-18] and the authors of [19] use sparse regression to learn population level ODEs from agent-based modeling simulations. The authors of [20] also derived a bias-correcting regression framework for inferring the drift and diffusion in underdamped Langevin dynamics, and in [21] a neural network-based algorithm for inferring SDEs was developed.

Only in the last few years have significant strides been made towards parameter inference of *interacting* particle systems such as (1.1) from data. Apart from some exceptions, such as a Gaussian process regression algorithm recently developed in [22], applications of maximum likelihood theory are by far the most frequently studied. An early but often overlooked work by Kasonga [23] extends the maximum-likelihood approach to inference of the interaction potential K, assuming full availability of the continuous particle trajectories and the diffusivity σ. Two decades later, Bishwal [24] further extended this approach to discrete particle observations in the specific context of linear particle interactions. In both cases, a sequence of finite-dimensional subspaces is used to approximate the interaction function, and convergence is shown as the dimension of the subspace J and number of particles N both approach infinity. More recently, the maximum likelihood approach has been carried out in [25,26] in the case of radial interactions and in [27] in the case of linear particle interactions and single-trajectory data (i.e. one instance of the particle system). The authors of [28] recently developed an online maximum likelihood method for inference of IPS, and in [29] maximum likelihood is applied to parameter estimation in an IPS for pedestrian flow. It should also be noted that parameter estimation for IPS is common in biological sciences, with the most frequently used technique being nonlinear least squares with a cost function comprised of summary statistics [7,30].

Problem (P) is made challenging by the coupled effects of K, V, and σ. In each of the previously mentioned algorithms, the assumption is made that σ is known and/or that K takes a specific form (radial or linear). In addition, the maximum likelihood-based approach approximates the differential $dX_{t}^{\left(i\right)}$ of particle i using a 1st-order finite difference: $dX_{t}^{\left(i\right)}\approx X_{t+\Delta t}^{\left(i\right)}-X_{t}^{\left(i\right)}$, which is especially ill-suited to problems involving *extrinsic* noise in the particle positions. Our primary goal is to show that the weak-form sparse regression framework allows for identification of the full model (K, V, σ), with significantly reduced computational complexity, when N is on the order of several thousands or more. We use a two-step process: the density of particles is approximated using a density kernel G and then the WSINDy algorithm (weak-form sparse identification of nonlinear dynamics) is applied in the PDE setting [2,3]. WSINDy is a modified version of the original SINDy algorithm [1,31] where the weak formulation of the dynamics is enforced using a family of test functions that offers reduced computational complexity, high-accuracy recovery in low-noise regimes, and increased robustness to high-noise scenarios. The feasibility of this approach for IPS is grounded in the convergence of IPS to associated mean-field equations. The reduction in computational complexity follows from the reduction in evaluation of candidate potentials (as discussed in Section 4.2), as well as the convolutional nature of the weak-form algorithm.

To the best of our knowledge, we present here the first *weak-form sparse regression* approach for inference of interacting particle systems, however we now review several related approaches that have recently been developed. In [32], the authors learn local hydrodynamic equations from active matter particle systems using the SINDy algorithm in the strong-form PDE setting. In contrast to [32], our approach learns nonlocal equations using the weak-form, however similarly to [32] we perform model selection and inference of parameters using sparse regression at the continuum level. The weak form provides an advantage because no smoothness is required on the particle density (for requisite smoothness the authors of [32] use a Gaussian kernel, which is more expensive to compute than simple particle binning as done here). The authors of [33] developed an integral formulation for inference of plasma physics models from PIC data using SINDy, however their method involves first computing strong-form derivatives and then averaging, rather than integration by parts against test functions as done here, and as in [32], the learned models are local. In [34], the authors apply the maximum likelihood approach in the continuum setting on the underlying nonlocal Fokker–Planck equation and learn directly the nonlocal PDE using strong-form discretizations of the dynamics. While we similarly use the continuum setting for inference (albeit in weak form), our approach differs from [34] in that it is designed for the more realistic setting of discrete-time *particle* data, rather than pointwise data on the particle *density* (assumed to be smooth in [34]).

### 2.1. Contributions

The purpose of the present article is to show that the weak form provides an advantage in speed and accuracy compared with existing inference methods for particle systems when the number of particles is sufficiently large (on the order of several thousand or more). The key points of this article include:

- Formulation of a weak-form sparse recovery algorithm for simultaneous identification of the particle interaction force K, local potential V, and diffusivity σ from discrete-time particle data.
- Convergence with rate $O(N^{-1∕2})$ of the resulting full-rank least-squares solution as the number of particles N→∞ and timestep Δt→0.
- Numerical illustration of (II) along with robustness to either intrinsic randomness (e.g. Brownian motion) or extrinsic randomness (e.g. additive measurement noise).

### 2.2. Paper organization

In Section 3 we review results from mean-field theory used to show convergence of the weak-form method. In Section 4 we introduce the WSINDy algorithm applied to interacting particles, including hyperparameter selection, computational complexity, and convergence of the method under suitable assumptions in the limit of large N. Section 5 contains numerical examples exhibiting the convergence rates of the previous section and examining the robustness of the algorithm to various sources of corruption, and Section 6 contains a discussion of extensions and future directions. In the Appendix we provide information on the hyperparameters used A.1, derivation of the homogenized equation (5.3) A.2, results and discussion for the case of small N and large M (in comparison with [26]) A.3, and proofs to technical lemmas A.4. Table 1 includes a list of notations used throughout.

## 3. Review of mean-field theory

Our weak-form approach utilizes that under fairly general assumptions the empirical measure $\mu_{t}^{N}$ of the process $X_{t}$ defined in (1.1) converges weakly to $\mu_{t}$, the distribution of the associated mean-field process $X_{t}$ defined in (3.2). Specifically, under suitable assumptions on V, K, σ, and $\mu_{0}$, there exists T>0 such that for all t∈[0,T], the mean-field limit^4^

$$\lim_{N\to \infty }\mu_{t}^{N}=\mu_{t}$$

holds in the weak topology of measures,^5^ where $\mu_{t}$ is a weak-measure solution to the mean-field dynamics

(3.1) $$\partial_{t}\mu_{t}=\nabla \cdot (\mu_{t}\;\nabla K*\mu_{t})+\nabla \cdot (\mu_{t}\;\nabla V)\;\;+\frac{1}{2}\sum_{i,j=1}^{d}\frac{\partial^{2}}{\partial x_{i}\partial x_{j}}(\sigma \sigma^{T}\mu_{t}),\;\;\mu_{0}\in P_{2}(R^{d}).$$

Eq. (3.1) describes the evolution of the distribution of the McKean–Vlasov process

(3.2) $$dX_{t}=-\nabla K*\mu_{t}\;(X_{t})\;dt-\nabla V\;(X_{t})\;dt+\sigma (X_{t})\;dB_{t}.$$

This implies that as N→∞, an initially correlated particle system driven by pairwise interaction becomes uncorrelated and only interacts with its mean-field distribution $\mu_{t}$. In particular, the following theorem summarizes several mean-field results taken from the review article [35] with proofs in [36,37].^6^

**Theorem** ([35-37]). *Assume that* ΔK *is globally Lipschitz*, V=0, *and* σ(x)=σ=const. *In addition assume that* $\mu_{0}\in P_{2}(R^{d})$. *Then for any* T>0, *for all* t≤T *it holds that*

- *There exists a unique solution* ($X_{t}$, $\mu_{t}$) *where* $X_{t}$ *is a strong solution to* (3.2) *and* $\mu_{t}$ *is a weak-measure solution to* (3.1).
- *For any* $\phi \in C_{b}^{1}(R^{d})$,
(3.3) $$E\left[{\left(\frac{1}{N}\sum_{i=1}^{N}\phi (X_{t}^{\left(i\right)})-\int_{R^{d}}\phi (x)d\mu_{t}(x)\right)}^{2}\right]\leq C\frac{\|\phi {\|}_{C^{1}}^{2}}{N}$$
  *with* C *depending on* Lip(∇K) *and* T.
- *For any* k∈N, *a.e.* −t<T, *the k-particle marginal*
$$\rho_{t}^{(k),N}(x_{1},\ldots ,x_{k})≔\int_{R^{d(N-k)}}F_{t}^{N}(x_{1},\ldots ,x_{k},x_{k+1},\ldots ,x_{N})\;\;\times dx_{k+1}\cdots dx_{N}$$
  *converges weakly to* $\mu_{t}^{⊗k}$ *as* N→∞, *where* $F_{t}^{N}\in P(R^{Nd})$ *is the distribution of* $X_{t}$.

The previous result immediately extends to the case of ∇V and σ both globally Lipschitz and has been extended to ∇K only locally-Lipschitz in [38], ∇K with Coulomb-type singularity at the origin in [39], and domains with boundaries in [40,41]. Analysis of the model (3.1) continues to evolve in various contexts, including analysis of equilibria [42-44] and connections to deep learning [45]. For our convergence result below we simply assume that $K^{⋆}$, $V^{⋆}$, $\sigma^{⋆}$ and $\mu_{0}$ are such that (*i*) and (*ii*) from the above theorem hold.

### 3.1. Weak form

Despite the $O(N^{-1∕2})$ convergence of the empirical measure in previous theorem, it is unclear at what particle number N the mean-field equations become a suitable framework for inference using particle data, due to the complex variance structure at any finite N. A key piece of the present work is to show that the weak form of the mean-field equations does indeed provide a suitable setting when N is at least several thousands. Moreover, since in many cases (3.1) can only be understood in a weak sense, the weak form is the natural framework for identification. We say that $\mu_{t}$ is a weak solution to (3.1) if for any $\psi \in C^{2}(R^{d}\times (0,T))$ compactly supported it holds that

(3.4) $$\int_{0}^{T}\int_{R^{d}}\partial_{t}\psi (x,t)\;d\mu_{t}(x)dt\;\;=\int_{0}^{T}\int_{R^{d}}\left(\nabla \psi (x,t)\cdot \nabla K*\mu_{t}(x)+\nabla \psi (x,t)\cdot \nabla V(x)\;\;-\frac{1}{2}\text{Tr}\left(\nabla^{2}\psi (x,t)\sigma (x)\sigma^{T}(x)\right)\right)\;d\mu_{t}(x)dt,$$

where $\nabla^{2}\psi$ denotes the Hessian of ψ and Tr(A) is the trace of the matrix A. Our method requires discretizing (3.4) for all ψ∈Ψ where $\Psi =(\psi_{1},\ldots ,\psi_{n})$ is a suitable test function basis, and approximating the mean-field distribution $\mu_{t}$ with a density $U_{t}$ constructed from discrete particle data at time t. We then find K, V, and σ within specified finite-dimensional function spaces.

## 4. Algorithm

We propose the general Algorithm 4.1 for discovery of mean-field equations from particle data. The inputs are a discrete-time sample Y containing M experiments each with N particle positions over L timepoints $t=(t_{1},\ldots ,t_{L})$. The following hyperparameters are defined by the user: (i) a kernel G used to map the empirical measure $\mu_{t}^{N}$ to an approximate density $U_{t}$, (ii) a spatial grid C over which to evaluate the approximate density $U_{t}=U_{t}(C)$, (iii) a library of trial functions $L=\{L_{K},L_{V},L_{\sigma }\}=\{(K_{j}{)}_{j=1}^{J_{K}},(V_{j}{)}_{j=1}^{J_{V}},(\sigma_{j}{)}_{j=1}^{J_{\sigma }}\}$, (iv) a basis of test functions $\Psi =(\psi_{k}{)}_{k=1}^{n}$, (v) a quadrature rule over the spatiotemporal grid (C, t) denoted by an inner product ⟨·, ·⟩, and (vi) sparsity factors λ for the modified sequential thresholding least-squares Algorithm 4.2 (MSTLS) reviewed below. We discuss choices of these hyperparameters in Section 4.1, computational complexity of the algorithm in Section 4.2, convergence of the algorithm in Section 4.3. In Section 4.4 we briefly discuss gaps between theory and practice. Table 1 includes a list of notations used throughout.

**Table:** 

Algorithm 4.1 WSINDy for identifying mean-field Eq. (3.1) from particle data Y $\left(\hat{w},\hat{\lambda })=\mathrm{WSINDy}\;(Y,t;\;G,\;C,\;L,\;\Psi ,\;\langle \cdot ,\cdot \rangle ,\;\lambda \right)$	


### 4.1. Hyperparameter selection

#### 4.1.1. Quadrature

We assume that the set of gridpoints C in Algorithm 4.1 is chosen from some compact domain $D\subset R^{d}$ containing supp (Y). The choice of C (and D) must be chosen in conjunction with the quadrature scheme, which includes integration in time using the given timepoints t as well as space. For completeness, the inner products in lines 10, 16, 22, and 27 of Algorithm 4.1 are defined in the continuous setting by

$$\langle f,g\rangle \;=\;\int_{0}^{T}\int_{D}f(x,t)g(x,t)dxdt\;,$$

and the convolution in line 10 is defined by

$$\nabla K_{j}*U_{t}(x)=\int_{D}\nabla K_{j}(x-y)U_{t}(y)dy.$$

In the present work we adopt the scheme used in the application of WSINDy for local PDEs [3], which includes the trapezoidal rule in space and time with test functions ψ compactly supported in D×(0,T). We take D to be a rectangular domain enclosing supp (Y) and C⊂D to be equally-spaced in order to efficiently evaluate convolution terms. In what follows we denote by ⟨·, ·⟨ the continuous inner product, 〈⋅,⋅〉 the inner product over $\langle \cdot ,\cdot \rangle_{h}$ evaluated using the composite trapezoidal rule in space with meshwidth D×[0,T] and Lebesgue integration in time, and by h the trapezoidal rule in both space and time, with meshwidth $\langle \cdot ,\cdot \rangle_{h,\Delta t}$ in space and h in time. With some abuse of notation, Δt will denote the convolution of f∗g and f, understood to be discrete or continuous by the context. Note also that we denote by g, $\mu^{N}$, and μ the measures over U defined by $R^{d}\times [0,T]$, $\mu_{t}^{N}\Lambda_{\left[0,T\right]}$ and $\mu_{t}\Lambda_{\left[0,T\right]}$, respectively, where $U_{t}\Lambda_{\left[0,T\right]}$ is the Lebesgue measure on [0, $\Lambda_{\left[0,T\right]}$].

#### 4.1.2. Density kernel

Having chosen the domain [0,t] containing the particle data $D\subset R^{d}$, let Y be a partition of $P^{h}=\{B_{k}{\}}_{k}$ with $D(\cup_{k}B_{k}=D)$ indicating the size of the atoms h. For the remainder of the paper we take $B_{k}$ to be hypercubes of equal side length $B_{k}$ in order to minimize computation time for integration, although this is by no means necessary. For particle positions h, we define the histogram^7^

(4.1) $$U_{t}=\sum_{k}\frac{1}{|B_{k}|}1_{B_{k}}(x)\left(\frac{1}{N}\sum_{i}1_{B_{k}}(X_{t}^{\left(i\right)})\right)=\int_{D}G(x,y)d\mu_{t}^{N}(y).$$

Here the *density kernel* is defined

$$G(x,y)=\sum_{k}\frac{1}{|B_{k}|}1_{B_{k}}(x)1_{B_{k}}(y),$$

and in this setting the corresponding spatial grid $X_{t}$ is the set of center-points of the bins $C=(c_{k}{)}_{k}$, from which we define the discrete histogram data $B_{k}$. The discrete histogram $U_{t}=U_{t}(C)$ then serves as an approximation to the mean-field distribution $U_{t}$.

Pointwise estimation of densities from samples of particles usually requires large numbers of particles to achieve reasonably low variance, and in general the variance grows inversely proportional to the bin width $\mu_{t}$. One benefit of the weak form is that integrating against a histogram h does not suffer from the same increase in variance with small U. In particular,

**Lemma 1.** *Let* h *be a sequence of* $\left(Y^{\left(1\right)},Y^{\left(2\right)},\ldots \right)$-*valued random variables such that the empirical measure* $R^{d}$ *of* $\mu^{N}$ *converges weakly to* $Y≔(Y^{\left(1\right)},\ldots ,Y^{\left(N\right)})$ *according to*

(4.2) $$E\left[(\langle \psi ,\mu^{N}\rangle -\langle \psi ,\mu \rangle {)}^{2}\right]\leq C\;\|\psi {\|}_{C^{1}}^{2}N^{-1}$$

*for all* $\mu \in p(R^{d})$ *and* $\psi \;\in \;C^{1}(R^{d})$ *a universal constant. Let* C *be the histogram computed with kernel* U *using* (4.1) *with* G *bins and equal sidelength* n. *Then for any* h *in* ψ *compactly supported in* $C^{1}(R^{d})$, *we have the mean-squared error (for* D *depending on* $\tilde{C}$ and C*)*

$$E\left[{\left(\langle \psi ,U\rangle_{h}-\langle \psi ,\mu \rangle \right)}^{2}\right]\leq \tilde{C}\;\|\psi {\|}_{C^{1}}^{2}\;(h^{2}+N^{-1}).$$

**Remark 1.** We note that (4.2) follows immediately for d i.i.d.,^8^ and also for $Y^{\left(i\right)}∼\mu$ a solution to (1.1) at time $Y=X_{t}$ with mean-field distribution t according to (3.3) (for suitable $\mu =\mu_{t}$, K, and V), which is the setting of the current article.

**Proof of** Lemma 1. First we note that by compact support of σ, the trapezoidal rule can be written

$$\langle \psi ,U\rangle_{h}={\langle \psi ,\int_{R^{d}}G(\cdot ,y)d\mu^{N}(y)\rangle }_{h}=\langle \psi^{C},\mu^{N}\rangle =\frac{1}{N}\sum_{i=1}^{N}\psi^{C}(Y^{\left(i\right)})$$

where the midpoint approximation ψ of $\psi^{C}$ is given by

(4.3) $$\psi^{C}(x)=\sum_{k=1}^{K}\psi (c_{k})1_{B_{k}}(x).$$

Hence we simply split the error and use (4.2):

$$E\left[(\langle \psi ,U\rangle_{h}-\langle \psi ,\mu \rangle {)}^{2}\right]\leq 2E\left[\langle \psi^{C}-\psi ,\mu^{N}\rangle^{2}\right]\;\;+2E\left[(\langle \psi ,\mu^{N}\rangle -\langle \psi ,\mu \rangle {)}^{2}\right]\;\;\leq \|\psi {\|}_{C^{1}}^{2}\left(\frac{d}{2}h^{2}+2CN^{-1}\right).$$

The previous lemma in particular shows that small bin width ψ does not negatively impact h as an estimator of $\langle \psi ,U\rangle_{h}$, which is in contrast to 〈ψ,μ〉 as a pointwise estimator of U(x). For example, if we assume that μ(x) is sampled from a Y density $C^{1}$, it is well known that the mean-square optimal bin width is μ [46]. Summarizing this result, elementary computation reveals the pointwise bias for $h=O(N^{-1∕3})$,

$$\text{bias}(U(x))=E\;[U(x)]-\mu (x)=\frac{\mu (B_{k})}{|B_{k}|}-\mu (x)≔\mu (\xi )-\mu (x)$$

for some $x\in B_{k}$. Letting $\xi \in B_{k}$, we have

$$\text{bias}(U(x){)}^{2}\leq L_{k}^{2}2^{d-1}h^{2}.$$

For the variance we get

$$\text{Var}(U(x))=\frac{1}{N}\frac{\mu (B_{k})(1-\mu (B_{k}))}{|B_{k}{|}^{2}}=\frac{\mu (\xi )}{N}\;(1-\mu (B_{k}))\frac{1}{{\sqrt{2}}^{d-1}h},$$

and hence a bound for the mean-squared error

$$E\left[(U(x)-\mu (x){)}^{2}\right]\leq L_{k}^{2}2^{d-1}h^{2}+\frac{\mu (\xi )}{N{\sqrt{2}}^{d-1}}h^{-1}.$$

Minimizing the bound over $L_{k}=\max_{x\in B_{k}}\;|\nabla \mu (x)|$ we find an approximately optimal bin width

$$h^{*}={\left(\frac{\rho (\xi )}{2^{\frac{3d-1}{2}}L_{k}^{2}}\right)}^{1∕3}N^{-1∕3}=O(N^{-1∕3}),$$

which provides an overall pointwise root-mean-squared error of h. Hence, not only does the weak form remove the inverse $O(N^{-1∕3})$ dependence in the variance, but fewer particles are needed to accurately approximate integrals of the density h.

#### 4.1.3. Test function basis

For the test functions μ we use the same approach as the PDE setting [3], namely we fix a *reference test function* $(\psi_{k}{)}_{1\leq k\leq n}$ and set

$$\psi_{k}(x,t)=\psi (x_{k}-x,t_{k}-t)$$

where ψ is a fixed set of *query points*. This, together with a separable representation

$$\psi (x,t)=\phi_{1}(x_{1})\cdots \phi_{d}(x_{d})\phi_{d+1}(t),$$

enables construction of the linear system ($Q\;≔\{(x_{k},t_{k}){\}}_{1\leq k\leq n}$, G) using the FFT. We choose b, $\phi_{j}$, of the form

(4.4) $$\phi_{m,p}(v;\;\Delta )≔\max{\left(1-{\left(\frac{v}{m\Delta }\right)}^{2},0\right)}^{p}$$

where 1≤j≤d+1 is the integer *support parameter* such that m is supported on $\phi_{m,p}$ points of spacing 2m+1 and Δ∈{h,Δt} is the *degree* of p≥1. For simplicity we set $\phi_{m,p}$ for $\phi_{j}=\phi_{m_{x},p_{x}}$ and 1≤j≤d, so that only the numbers $\phi_{d+1}=\phi_{m_{t},p_{t}}$, $m_{x}$, $p_{x}$, $m_{t}$ need to be specified.

Since $p_{t}$ has exactly $\phi_{m,p}$ weak derivatives, p and $p_{x}$ must be at least as large as the maximum spatial and temporal derivatives appearing in the library $p_{t}$, or L, $p_{x}\geq 2$. Larger $p_{t}\geq 1$ results in higher-accuracy enforcement of the weak form (3.4) in low-noise situations (see Lemma 2 of [2] for details), however the convergence analysis below indicates that smaller p, $\text{Lip}(\partial^{\alpha }\psi )$, may reduce variance. The support parameter m determines the length and time scales of interest and must be chosen small enough to extract relevant scales yet large enough to sufficiently reduce variance.

In [3, Appendix A] the authors developed a changepoint algorithm to choose ∣α∣≤2, $m_{x}$, $m_{t}$, $p_{x}$ automatically from the Fourier spectrum of the data $p_{t}$. Here, for each of the three examples in Section 5, we fix U across all particle numbers ψ, extrinsic noises N, and intrinsic noises ε, in order to instead focus on convergence in σ. To strike a balance between accuracy and small N we choose Lip(ψ) and $p_{t}=3$ throughout. We used a combination of the changepoint algorithm and manual tuning to arrive at $p_{x}=5$ and $m_{x}$ which work well across all noise levels and numbers of particles examined. Query points $m_{t}$ are taken to be an equally-spaced subgrid of Q with spacing C and $s_{x}$ for spatial and temporal coordinates. The resulting values $s_{t}$, $p_{x}$, $p_{t}$, $m_{x}$, $m_{t}$, and $s_{x}$ determine the weak discretization scheme and can be found in Appendix A.1 for each example below.

The results in Section 5 appear robust to $s_{t}$, $3\leq p_{x}$. In addition, choosing $p_{t}\leq 9$ and $m_{x}$ specific to each dataset $m_{t}$ using the changepoint method often improves results. Although automated in the changepoint algorithm, we recommend visualizing the overlap between the Fourier spectra of Y and ψ when choosing U, $p_{x}$, $p_{t}$, $m_{x}$ in order to directly observe which the modes in the data will experience filtering under convolution with $m_{t}$. In general, there is much flexibility in the choice of ψ. Optimizing ψ continues to be an active area of research.

#### 4.1.4. Trial function library

The general Algorithm 4.1 does not impose a radial structure for the interaction potential ψ, nor does it assume any prior knowledge that the particle system is in fact interacting. In the examples below,^9^ the libraries K, $L_{K}$, $L_{V}$ are composed of monomial and/or trigonometric terms to demonstrate that sparse regression is effective in selecting the correct combination of nonlocal drift, local drift, and diffusion terms. Rank deficiency can result, however, from naive choices of nonlocal and local bases. Consider the kernel $L_{\sigma }$, which satisfies

$$\nabla K*\mu_{t}=x-M_{1}(\mu_{t})=\nabla V(x)$$

where $K(x)=\frac{1}{2}|x{|}^{2}$ and $V(x)=\frac{1}{2}|x-M_{1}(\mu_{t}){|}^{2}$ is the first moment of $M_{1}(\mu_{t})$. Since $\mu_{t}$ is conserved in the model (3.2) posed in free-space,^10^ including the same power-law terms in both libraries $M_{1}(\mu_{t})$ and $L_{K}$ will lead to rank deficiency. This is easily avoided by incorporating known symmetries of the model (3.2), however in general we recommend that the user builds the library $L_{V}$ incrementally and monitors the condition number of L while selecting terms.

#### 4.1.5. Sparse regression

As in [3], we enforce sparsity using a *modified* sequential thresholding least-squares algorithm (MSTLS), included as Algorithm 4.2, where the “modifications” are two-fold. First, we incorporate into the thresholding step the magnitude of the overall term G as well as the coefficient magnitude ${\left\|w_{j}G_{j}\right\|}_{2}$, by defining non-uniform lower and upper thresholds

(4.5) {Ljλ=λmax{1,‖b‖‖Gj‖}Ujλ=1λmin{1,‖b‖‖Gj‖}},1≤j≤J,

where $|w_{j}|$ is the number of columns in $J=J_{K}+J_{V}+J_{\sigma }$. Second, we perform a grid search^11^ over candidate sparsity parameters G and choose the parameter λ that is the smallest minimizer over $\hat{\lambda }$ of the cost function

(4.6) $$L(\lambda )=\frac{\|G(w^{\lambda }-w^{0}){\|}_{2}}{\|{\mathrm{Gw}}^{0}{\|}_{2}}+\frac{\|w^{\lambda }{\|}_{0}}{J}$$

where λ is the output of the sequential thresholding algorithm with non-uniform thresholds (4.5) and $w^{\lambda }$ is the least-squares solution.^12^ The final coefficient vector is then set to $w^{0}=G^{†}b$.

We now review some aspects of Algorithm 4.2. Results from [47] on the convergence of STLS carry over for the inner loop of Algorithm 4.2, namely if $\hat{w}=w^{\hat{\lambda }}$ is full-rank, the inner loop terminates in at most G iterations with a resulting coefficient vector J that is a local minimizer of the cost function $w^{\lambda }$. This implies that the full algorithm terminates in atmost $F(w)=\|\mathrm{Gw}-b{\|}_{2}^{2}+\lambda^{2}\;\|w{\|}_{0}$ least-squares solves (each on a subset of columns of mJ).

When considering recovery of the true weight vector G, Theorem 1 implies convergence in particle number $w^{⋆}$ of N to $\hat{w}$ when $w^{⋆}$ is full-rank. The rate of convergence depends implicitly on the condition number of G, hence it is recommended that one builds the library G incrementally, stopping before the conditional number L grows too large. If κ(G) is rank deficient, classical recovery guarantees from compressive sensing do not necessarily apply, due to high correlations between the columns of G (recall each column is constructed from the same dataset G).^13^ One may employ additional regularization (e.g. Tikhonov regularization as in [31]); however, in general, improvements to existing sparse regression algorithms for rank-deficient, noisy, and highly-correlated matrices is an active area of research.

**Table:** 

Algorithm 4.2 Modified sequential thresholding with automatic threshold selection U	


The bounds (4.5) enforce a quasi-dominant balance rule, such that ${\left\|w_{j}G_{j}\right\|}_{2}$ is within $\log_{10}(\lambda )$ orders of magnitude from $\|b{\|}_{2}$ and $|w_{j}|$ is within $\log_{10}(\lambda )$ orders of magnitude from 1 (the coefficient of time derivative $\partial_{t}\mu_{t}$. This is specifically designed to handle poorly-scaled data (see the Burgers and Korteweg–de Vries examples in [3]), however we leave a more thorough examination of the thresholding requirements necessary for models with multiple scales to future work.

As the sum of two relative errors, minimizers of the cost function L equally weight the accuracy and sparsity of $w^{\hat{\lambda }}$. By choosing $\hat{\lambda }$ to be the smallest minimizer of L over λ, we identify the thresholds λ∈λ such that $\lambda <\hat{\lambda }$ as those resulting in an overfit model. We commonly choose λ to be log-equally spaced (e.g. 50 points from 10^−4^ to 1), and starting from a coarse grid, refine λ until the minimum of L is stationary.

### 4.2. Computational complexity

To compute convolutions against ∇K for each $K\in L_{K}$, we first evaluate $(\partial_{x_{i}}K{)}_{1\leq i\leq d}$ at the grid C−C defined by

$$C-C≔\{x\in R^{d}\;:\;x=(i_{1}h,\ldots ,i_{d}h),\;\;-n_{\ell }\leq i_{\ell }\leq n_{\ell }\},$$

where h is the spacing of C and $n_{\ell }$, 1≤ℓ≤d, is the number of points in C along the ℓth coordinate. Computing^14^ $\partial_{x_{i}}K\;≔\partial_{x_{i}}(C-C)$ requires $2^{d}|C|$ evaluations of K, where $|C|=\prod_{\ell =1}^{d}n_{\ell }$ is the number of points in C. We then use the d-dimensional FFT to compute the convolutions

$$\partial_{x_{i}}K*U_{t}\approx \partial_{x_{i}}K*U_{t}(C),\;\;t\in t$$

where only entries corresponding to particle interactions within C are retained. For d=1 this amounts to O(∣C∣log∣C∣) flops per timestep. For d=2 and higher dimensions, the d-dimensional FFT is considerably slower unless one of the arrays is separable. To enforce separability, trial interaction potentials in $L_{K}$ can be chosen to be a sum of separable functions,

(4.7) $$K(x)=\sum_{q=1}^{Q}k_{1,q}(x_{1})\cdots k_{d,q}(x_{d}),$$

in which case only a series of one-dimensional FFTs are needed to compute $\partial_{x_{i}}K*U_{t}$, and again the cost is O(∣C∣log∣C∣) per timestep. When K is not separable, a low-rank approximation can be computed from $\partial_{x_{i}}K$,

(4.8) $$\partial_{x_{i}}K\approx \sum_{q=1}^{Q}\sigma_{q}k_{1,q}⊗\cdots ⊗k_{d,q}$$

which again reduces convolutions to a series of one-dimensional FFTs. For d=2, this is accomplished using the truncated SVD, while for higher dimensions there does not exist a unique *best* rank-Q tensor approximation, although several efficient algorithms are available to compute a sufficiently accurate decomposition [49-51] (and the field of fast tensor decompositions is advancing rapidly).

We propose to compute convolutions by first computing a low-rank decomposition of $\partial_{x_{i}}K$ using the randomized truncated SVD [52] or a suitable randomized tensor decomposition and then applying the d-dimensional FFT as a series of one-dimensional FFTs. In the examples below we consider only d=1 and d=2, and leave extension to higher dimensions to future work.

Using low-rank approximations, the mean-field approach provides a significant reduction in computational complexity compared to direct evaluations of particle trajectories when N is sufficiently large. A particle-level computation of the nonlocal force in weak-form requires evaluating terms of the form

$$\sum_{\ell =1}^{L}\left(\frac{1}{N^{2}}\sum_{i=1}^{N}\sum_{j=1}^{N}\partial_{x}\psi (X_{t_{\ell }}^{\left(i\right)},t_{\ell })\partial_{x}K(X_{t_{\ell }}^{\left(i\right)}-X_{t_{\ell }}^{\left(j\right)})\right)\Delta t\;\;={\langle \partial_{x}\psi ,\mu^{N}(\partial_{x}K*\mu^{N})\rangle }_{h,\Delta t}.$$

**Fig. 1.**
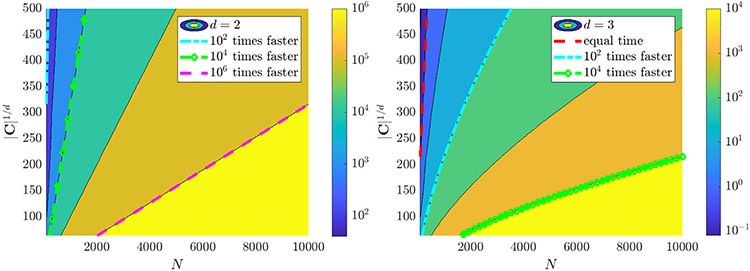
Factor by which the mean-field evaluation of interaction forces using histograms reduces total function evaluations as a function of particle number N and average gridpoints per dimension ${|C|}^{1∕d}$ for data with M=10 experiments each with L=100 timepoints. For example, with d=2 spatial dimensions (left) and N>2000 particles, the number of function evaluations is reduced by at least a factor of 10^4^.

For a single candidate interaction potential K, a collection of J test functions ψ, and M experiments, this amounts to $MLN^{2}+MLNJ$ function evaluations in $R^{d}$ and $O(MLN^{2}J)$ flops. If we use the proposed method, employing the convolutional weak form with a separable reference test function ψ (as in WSINDy for PDEs [3]) and exploiting a rank Q approximation of $\partial_{x}K$ when computing convolutions against interaction potential, we instead evaluate

$$\partial_{x}\psi *(U(\partial_{x}K*U))$$

using O(LQ∣C∣log(∣C∣)) flops and only $2^{d}|C|$ evaluations of $\partial_{x}K$, reused at each of the L timepoints.^15^ Fig. 1 provides a visualization of the reduction in function evaluations for L=100 timepoints and M=10 experiments over a range of N and $|C{|}^{1∕d}$ (points along each spatial dimension when ∣C∣ is a hypercube) in d=2 and d=3 spatial dimensions. Table 5 in Appendix A.1 lists walltimes for the examples below, showing that with N=64,000 particles the full algorithm implemented in MATLAB runs in under 10 s with all computations in serial on a laptop with an AMD Ryzen 7 pro 4750u processor, and requiring less than 8 Gb of RAM. The dependence on N is only through the O(N) computation of the histogram, hence this approach may find applications in physical coarse-graining (e.g. of molecular dynamics or plasma simulations).

### 4.3. Convergence

We now show that the estimators $\hat{K}$, $\hat{V}$, and $\hat{\sigma }$ of the weak-form method converge with a rate $O(h+N^{-1∕2}+\Delta t^{\eta })$ when ordinary least squares are used (i.e. λ=0) and only M=1 experiment is available. Here η>0 is the Hölder exponent of the sample paths of the process $X_{t}$. We assume that D, C, G, $P^{h}$ and the resulting histogram $U=(U_{t}{)}_{t\leq T}$ are as in Section 4.1.2. We make the following assumptions on the true model and resulting linear system throughout this section.

**Assumption H.** Let p≥1 be fixed.

(H.1) For each N≥2, $X_{t}=(X_{t}^{\left(1\right)},\ldots ,X_{t}^{\left(N\right)})$ is a strong solution to (1.1) for t∈[0,T], and for some η>0 the sample paths $t\to X_{t}^{\left(i\right)}(\omega )$ are almost-surely η-Hölder continuous, i.e. for some $C_{\eta }>0$,
$$|X_{t}^{\left(i\right)}(\omega )-X_{s}^{\left(i\right)}(\omega )|\leq C_{\eta }|t-s{|}^{\eta },\;\;\forall \;0\leq s\leq t\leq T,\;\forall \;1\leq i\leq N,\;\;\text{for}\;\text{a.e.}\;\omega \in \Omega .$$
(H.2) The initial particle distribution $\mu_{0}$ satisfies the moment bound
$$\int_{R^{d}}|x{|}^{p}d\mu_{0}(x)≔M_{p}<\infty .$$
(H.3) $\nabla K^{⋆}$ and $\nabla V^{⋆}$ satisfy for some $C_{p}>0$ the growth bound:
$$|\nabla V^{⋆}(x)-\nabla V^{⋆}(y)|+|\nabla K^{⋆}(x)-\nabla K^{⋆}(y)|\;\;\leq C_{p}|x-y|(1+\max\{|x|,|y|{\}}^{p-1}),\;\;x,y\in R^{d}.$$
(H.4) For the same constant $C_{p}>0$, it holds that^16^
$$\|\sigma^{⋆}(x)-\sigma^{⋆}(y){\|}_{F}\leq C_{p}|x-y{|}^{1∕2}\;\;\times (1+\max\{|x|,|y|{\}}^{p∕2-1∕2}),\;\;x,y\in R^{d}$$
(H.5) The test functions $\left(\psi_{k}{)}_{1\leq k\leq \eta }\subset C^{2}(R^{d}\times (0,T)\right)$ are compactly supported and together with the library L are such that G has full column rank with^17^ ${\left\|G^{†}\right\|}_{1}\leq C_{G}$ almost surely for some constant $C_{G}>0$.
(H.6) The true functions $K^{⋆}$, $V^{⋆}$, and $\sigma^{⋆}$ are in the span of L.

We will now define some notation and state some technical lemmas with proofs found in Appendix A.4. Define the weak-form operator

(4.9) $$L(\rho ,\psi ,\langle \cdot ,\cdot \rangle )\;≔\langle \partial_{t}\psi -\nabla \psi \cdot \nabla K^{⋆}*\rho -\nabla \psi \cdot \nabla V^{⋆}+\frac{1}{2}\text{Tr}(\nabla^{2}\psi \sigma^{⋆}(\sigma^{⋆}{)}^{T}),\rho \rangle ,$$

where $\rho \;=\;(\rho_{t}{)}_{t\leq T}$ is a curve in $P_{p}(R^{d})$, ψ is a $C^{2}$ function compactly supported over $R^{d}\times (0,T)$, and 〈⋅,⋅〉 is an inner product over $R^{d}\times (0,T)$. If $\rho =(\mu_{t}{)}_{t\leq T}$ is a weak solution to (3.1) and 〈⋅,⋅〉 is the $L^{2}(R^{d})$ inner product then L(ρ,ψ,〈⋅,⋅〉)=0. If instead $\rho =(\mu_{t}^{N}{)}_{t\leq T}$, then by Itô’s formula L(ρ,ψ,〈⋅,⋅〉) takes the form of an Itô integral, and we have the following:

**Lemma 2.** *Under* Assumptions (H.1)-(H.5)*, there exists a constant* C>0 *independent of* N *such that*

$$E\left[|L(\mu^{N},\psi ,\langle \cdot ,\cdot \rangle )|\right]\leq \frac{C}{\sqrt{N}}.$$

**Proof.** See Appendix A.4.

With the following lemma, we can relate the histogram U to the empirical measure $\mu^{N}$ through L using the inner product $\langle \cdot ,\cdot \rangle_{h}$ defined by trapezoidal-rule integration in space and continuous integration in time.

**Lemma 3.** *Under* Assumptions (H.1)-(H.5)*, for* C *independent of* N *and* h*, it holds that*

$$E\left[|L(U,\psi ,\langle \cdot ,\cdot \rangle_{h})-L(\mu^{N},\psi ,\langle \cdot ,\cdot \rangle )|\right]\leq Ch.$$

**Proof.** See Appendix A.4.

To incorporate discrete-time effects, we consider the difference between $L(U,\psi ,\langle \cdot ,\cdot \rangle_{h})$ and $L(U,\psi ,\langle \cdot ,\cdot \rangle_{h,\Delta t})$, where recall that $\langle \cdot ,\cdot \rangle_{h,\Delta t}$ denotes trapezoidal rule integration in space with meshwidth h and in time with sampling rate Δt.

**Lemma 4.** *Under* Assumptions (H.1)-(H.5)*, for* C *independent of* N, h*, and* Δt*, it holds that*

$$E\left[|L(U,\psi ,\langle \cdot ,\cdot \rangle_{h})-L(U,\psi ,\langle \cdot ,\cdot \rangle_{h,\Delta t})|\right]\leq C(h+\Delta t^{\eta }).$$

**Proof.** See Appendix A.4.

The previous estimates directly lead to the following bound on the model coefficients $\hat{w}$:

**Theorem 1.** *Assume that Assumption* H *holds. Let* $\hat{w}$ *be the learned model coefficients and* $w^{⋆}$ *the true model coefficients. For* C *independent of* N, h*, and* Δt *it holds that*

$$E\left[{\left\|\hat{w}-w^{*}\right\|}_{1}\right]\leq C\left(h+N^{-1∕2}+\Delta t^{\eta }\right).$$

**Proof.** Using that $K^{⋆}$, $V^{⋆}$, and $\sigma^{⋆}$ are in the span of L (H.6), we have that

$$b_{k}=\langle \partial_{t}\psi_{k},U\rangle_{h,\Delta t}=L(U,\psi_{k},\langle \cdot ,\cdot \rangle_{h,\Delta t})+G_{k}^{T}w^{⋆}≔L_{k}+G_{k}^{T}w^{⋆},$$

where $G_{k}^{T}$ is the kth row of G. From Lemmas 2-4 we have

$$E\;[|L_{k}|]\leq E\left[|L(U,\psi_{k},\langle \cdot ,\cdot \rangle_{h,\Delta t})-L(U,\psi_{k},\langle \cdot ,\cdot \rangle_{h})|\right]\;+E\left[|L(U,\psi_{k},\langle \cdot ,\cdot \rangle_{h})-L(\mu^{N},\psi_{k},\langle \cdot ,\cdot \rangle )|\right]\;+E\left[L(\mu^{N},\psi_{k},\langle \cdot ,\cdot \rangle )|\right]\;\leq C^{\prime }\;(h+N^{-1∕2}+\Delta t^{\eta }).$$

Using that G is full rank, it holds that $\hat{w}=G^{†}b=G^{†}L+w^{⋆}$, hence the result follows from the uniform bound on ${\left\|G^{†}\right\|}_{1}$ (H.5):

$$E\;[\|\hat{w}-w^{⋆}{\|}_{1}]\leq E\;[\|G^{†}{\|}_{1}\|L{\|}_{1}]\leq C^{\prime }C_{G}\;(h+N^{-1∕2}+\Delta t^{n}).$$

□

Under the assumption (H.6), an immediate corollary is

(4.10) E[‖K⋆−K^‖L2(D−D)+‖V∗−V^‖L2(D)][+‖‖σ⋆(σ⋆)T−σ^(σ^)T‖F‖L2(D)]≤C(h+N−1∕2+Δtη),

This follows from

$$\|K^{⋆}-\hat{K}{\|}_{L^{2}(D-D)}\leq \sum_{j=1}^{J}|w_{j}^{⋆}-{\hat{w}}_{j}|\;\|K_{j}{\|}_{L^{2}(D-D)}\;\;\leq \left(\underset{j}{\text{sup}}\|K_{j}{\|}_{L^{2}(D-D)}\right)\|w^{⋆}-\hat{w}{\|}_{1},$$

and similarly for $\hat{V}$ and $\hat{\sigma }$. Finally, setting $h=N^{-\alpha }$ for α>0 will ensure convergence as N→∞ and Δt→0.

### 4.4. Theory vs. Practice

We now make several remarks about the practical performance of Algorithm 4.1 with respect to the theoretical convergence of Theorem 1.

**Remark 2.** An important case of Theorem 1 is $\sigma^{⋆}\;=\;0$, in which case $\mu_{t}^{N}$ itself is a weak-measure solution to the mean-field Eq. (3.1) and the algorithm returns, for η≥2, $\left\|\hat{w}-w^{⋆}{\|}_{1}\leq C(h+\Delta t^{\eta }\right)$. This partially explains the accuracy observed for purely-extrinsic noise examples in Figs. 5 and 9. We note further that in the absence of noise (ε=0 and $\sigma^{⋆}=0$, not included in this work) Algorithm 4.1 recovers systems to high accuracy similarly to WSINDy applied to local dynamical systems [2,3].

**Remark 3.** Algorithm 4.1 in general implements sparse regression, yet Theorem 1 deals with ordinary least squares. Since least squares is a common subroutine of many sparse regression algorithms (including the MSTLS algorithm used here), the result is still relevant to sparse regression. Lastly, the full-rank assumption on G implies that as N→∞ sequential thresholding reduces to least squares.

**Remark 4.** Theorem 1 assumes data from a single experiment (M=1), while the examples below show that M>1 experiments improve results. For any fixed M>1, the N→∞ limit results in convergence, however, the N-fixed and M→∞ limit does not result in convergence, as this does not lead to the mean-field equations.^18^ The examples below show that using M>1 has a practical advantage, and in Appendix A.3 we demonstrate that even for small particle systems (N=10) the large M regime yields satisfactory results.

**Remark 5.** Many interesting examples have non-Lipschitz ∇K, in particular a lack of smoothness at x=0. If $\mu_{t}^{N}$ does not converge to a singular measure as N→∞, then the bound (A.4) holds for ∇K with a jump discontinuity at x=0, where an additional O(h) term arises from pairwise interactions within an O(h) distance. The examples below are chosen in part to show that $O(N^{-1∕2})$ convergence holds for ∇K with jumps at the origin.

## 5. Examples

We now demonstrate the successful identification of several particle systems in one and two spatial dimensions as well as the $O(N^{-1∕2})$ convergence predicted in Theorem 1. In each case we use Algorithm 4.1 to discover a mean-field equation of the form (3.1) from discrete-time particle data. For each dataset we simulate the associated interacting particle system $X_{t}$ given by (1.1) using the Euler–Maruyama scheme (initial conditions and timestep are given in each example). We assess the ability of WSINDy to select the correct model using the *true positivity ratio*^19^

(5.1) $$\text{TPR}(\hat{w})=\frac{\text{TP}}{\text{TP}+\text{FN}+\text{FP}}$$

where TP is the number of correctly identified nonzero coefficients, FN is the number of coefficients falsely identified as zero, and FP is the number of coefficients falsely identified as nonzero [53]. To demonstrate the $O(N^{-1∕2})$ convergence given by (4.10), for correctly identified models (i.e. $\text{TPR}(\hat{w})=1$) we compute the relative $\ell_{2}$-error of the recovered interaction force $\nabla \hat{K}$, local force $\nabla \hat{V}$, and diffusivity $\hat{\sigma }$ over C−C and C, respectively, denoting this by ‖⋅‖ in the plots below. Results are averaged over 100 trials.

For the computational grid C we first compute the sample standard deviation s of Y and we choose D to be the rectangular grid extending 3s from the mean of Y in each spatial dimension. We then set C to have 128 points in x and y for d=2 dimensions, and 256 points in x for d=1, noting that these numbers are fairly arbitrary, and used to show that the grid need not be too large. We set the sparsity factors so that $\log_{10}(\lambda )$ contains 100 equally spaced points from −4 to 0. More information on the specifications of each example can be found in Appendix A.1. (MATLAB code used to generate examples is available at https://github.com/MathBioCU/WSINDy_IPS.)

### 5.1. Two-dimensional local model and homogenization

The first system we examine is a local model $\left(K^{⋆}(x,y)=0\right)$ defined by the local potential $V^{⋆}(x,y)=-x-y$ and diffusivity $\sigma^{⋆}(x,y)=\sqrt{2\;(1+0.95\;cos(\omega x)\;cos(\omega y))}I_{2}$, where $I_{2}$ is the identity in $R^{2}$. This results in a constant advection, variable diffusivity mean-field model^20^

(5.2) $$\partial_{t}\mu_{t}=-\partial_{x}\mu_{t}-\partial_{y}\mu_{t}+\Delta \;[(1+0.95\;\cos(\omega x)\;\cos(\omega y))\;\mu_{t}].$$

The purpose of this example is three-fold. First, we are interested in the ability of Algorithm 4.1 to correctly identify a local model from a library containing both local and nonlocal terms. Next, we evaluate whether the $O(N^{-1∕2})$ convergence is realized. Lastly, we investigate whether for large ω the weak-form identifies the associated homogenized equation (derived in Appendix A.2)

(5.3) $$\partial_{t}\mu_{t}=-\partial_{x}\mu_{t}-\partial_{y}\mu_{t}+\bar{\omega }\Delta \mu_{t},$$

where $\bar{\omega }$ is given by the harmonic mean of diffusivity:

$$\bar{\omega }={\left(\int_{D}\frac{dxdy}{1+0.95\;\cos(x)\;\cos(y)}\right)}^{-1}.$$

**Fig. 2.**
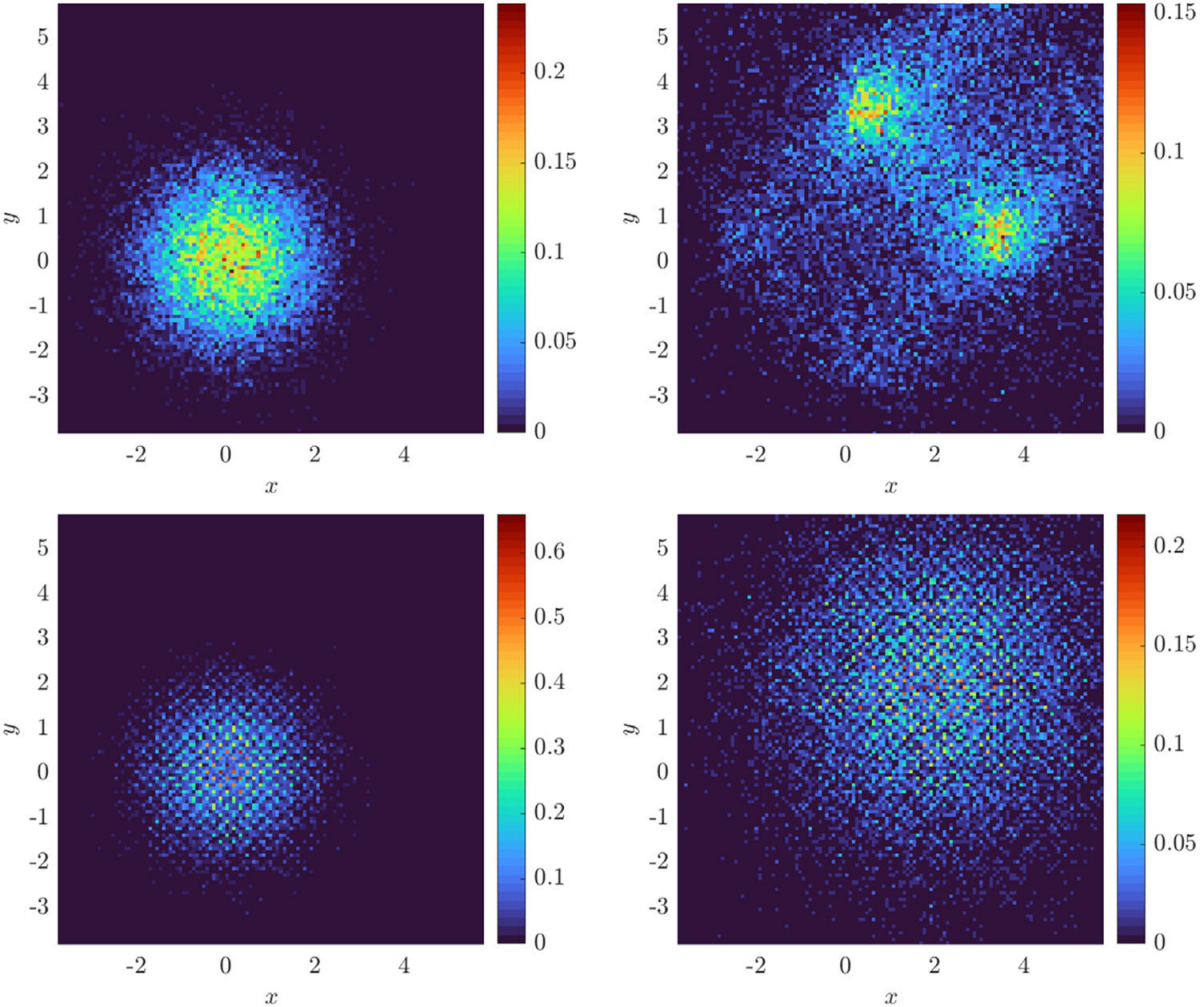
Snapshots at time t=2Δt=0.06 (left) and t=100Δt=2 (right) of histograms computed with 128 bins in x and y from 16,384 particles evolving under (5.2) with ω=1 (top) and ω=20 (bottom).

**Fig. 3.**
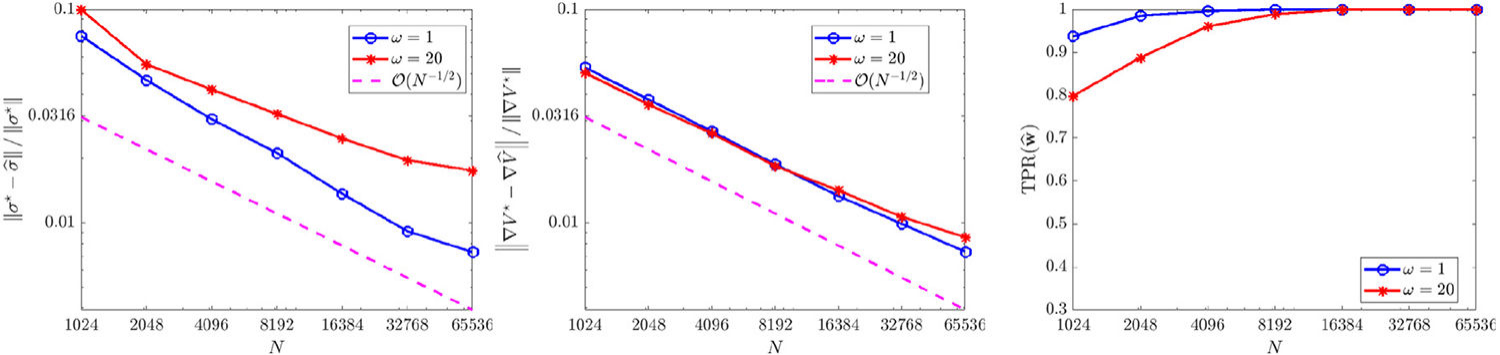
Convergence of $\hat{\sigma }$ (left) and $\nabla \hat{V}$ (middle), recall ‖⋅‖ denotes the $\ell_{2}$ norm, for (5.2) with ω∈{1,20}, as well as $\mathrm{TPR}(\hat{w})$ (right). For ω=1, results are compared to the exact model (5.2), while for ω=20 results are compared to the homogenized equation (5.3).

For ω∈{1,20} we evolve the particles from an initial Gaussian distribution with mean zero and covariance $I_{2}$ and record particle positions for 100 timesteps with Δt=0.02 (subsampled from a simulation with timestep 10^−4^). We use a rectangular domain D of approximate sidelength 10 and compute histograms with 128 bins in x and y for a spatial resolution of Δx≈0.078 (see Fig. 2 for solution snapshots), over which $\bar{\omega }\approx 0.62$. For ω=1 we compare recovered equations with the full model (5.2), while for ω=20 we compare with (5.3), for comparison computing $\bar{\omega }$ over each domain D using MATLAB’s integral2. Fig. 3 shows that as the particle number increases, we do in fact recover the desired equations, with $\text{TPR}(\hat{w})$ approaching one as N increases. For ω=1 we observe $O(N^{-1∕2})$ convergence of the local potential $\hat{V}$ and the diffusivity $\hat{\sigma }$. For ω=20, we observe approximate $O(N^{-1∕2})$ convergence of $\hat{V}$, and $\hat{\sigma }$ converging to within 2% of $\sqrt{2\bar{\omega }}$, the homogenized diffusivity (higher accuracy can hardly be expected for ω=20 since (5.3) is itself an approximation in the limit of infinite ω).

### 5.2. One-dimensional nonlocal model

We simulate the evolution of particle systems under the quadratic attraction/Newtonian repulsion potential 1 2

(5.4) $$K_{\text{QANR}}(x)=\frac{1}{2}x^{2}-|x|$$

with no external potential (V=0). The −∣x∣ portion of $K_{\text{QANR}}$, leading to a discontinuity in ∇K, is the one-dimensional free-space Green’s function for −Δ. For d≥1, when replaced by the corresponding Green’s function in d dimensions, the distribution of particles evolves under $K_{\text{QANR}}$ into the characteristic of the unit ball in $R^{d}$, which has implications for design and control of autonomous systems [54]. We compare three diffusivity profiles, σ(x)=0 corresponding to zero intrinsic noise, $\sigma (x)=\sqrt{2(0.1)}$ leading to constant-diffusivity intrinsic noise, and $\sigma (x)=\sqrt{2(0.1)}|x-2|$ leading to variable-diffusivity intrinsic noise. With zero intrinsic noise (σ(x)=0), we examine the effect of extrinsic noise on recovery, and assume uncertainty in the particle positions due to measurement noise at each timestep, Y=X+ε, for $\varepsilon ∼N(0,\epsilon^{2}\;\|X_{t}{\|}_{\text{RMS}}^{2})$ i.i.d. and ϵ∈ {0.01, 0.0316, 0.1, 0.316}. In this way ϵ is the *noise ratio*, such that $\|\varepsilon {\|}_{F}\;∕\;\|X{\|}_{F}\approx \epsilon$ (computed with ε and X stretched into column vectors).

**Fig. 4.**
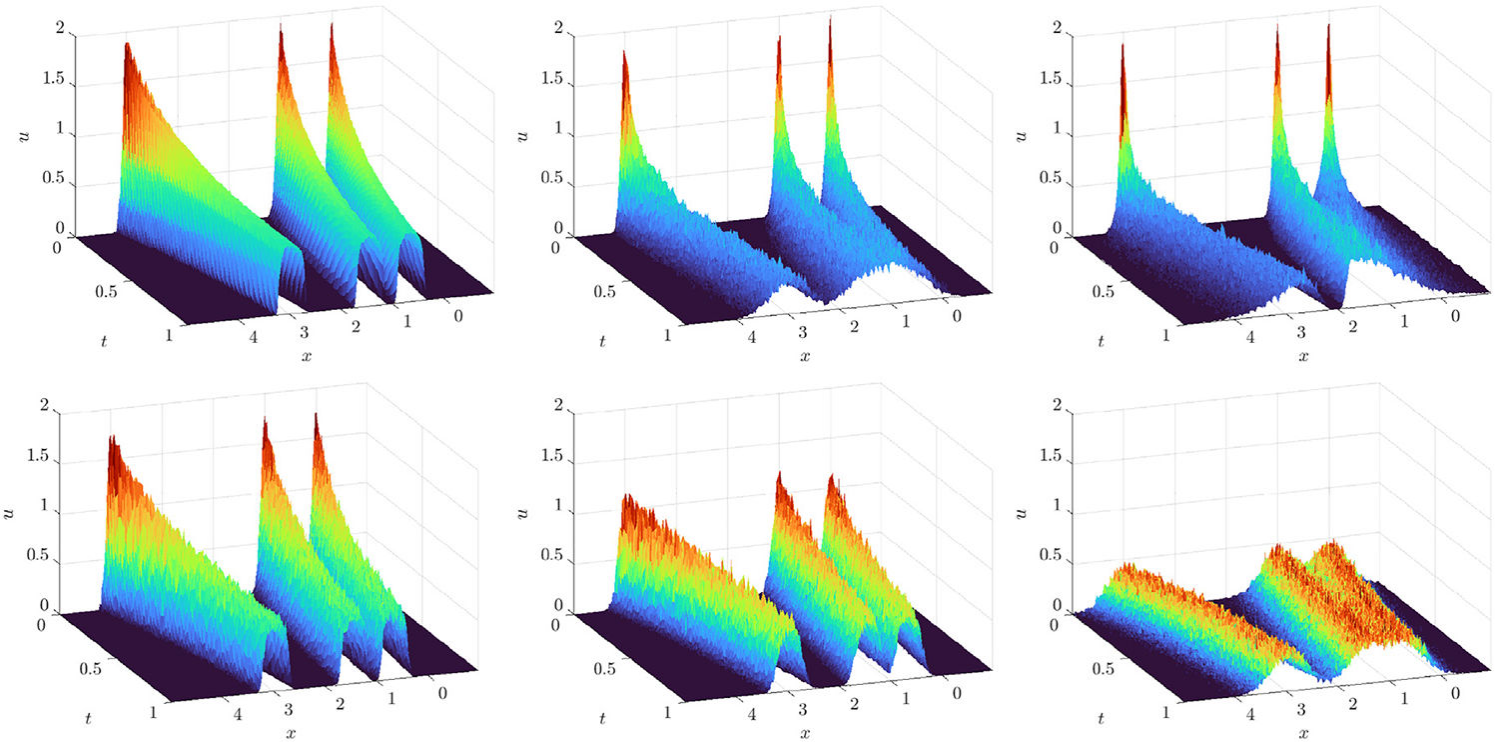
Histograms computed with 256 bins width h=0.0234 from 8000 particles in 1D evolving under $K^{⋆}=K_{\mathrm{QANR}}(x)$ (5.4). Top left to top right: $\sigma^{⋆}=0$, $\sigma^{⋆}(x)=\sqrt{2(0.1)}$, $\sigma^{⋆}(x)=\sqrt{2(0.1)}|x-2|$. Bottom: deterministic particles with i.i.d. Gaussian noise added to particle positions with resulting noise ratios (left to right) ϵ=0.0316, 0.1, 0.316.

Measurement data consists of 100 timesteps at resolution Δt=0.01, coarsened from simulations with timestep 0.001. Initial particle positions are drawn from a mixture of three Gaussians each with standard deviation 0.005. Histograms are constructed with 256 bins of width h=0.0234. Typical histograms for each noise level are shown in Fig. 4 computed one experiment with N=8000 particles.

**Fig. 5.**
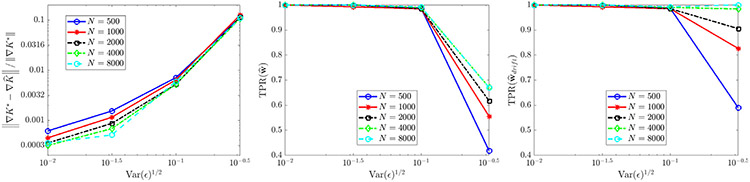
Recovery of (3.1) in one spatial dimension for $K^{⋆}=K_{\mathrm{QANR}}$ and $\sigma^{⋆}=0$ under different levels of observational noise ϵ. Left: relative error in learned interaction kernel $\hat{K}$. Middle: true positivity ratio for full model (3.1). Right: true positivity ratio for drift term.

For the case of extrinsic noise (Fig. 5), we use only one experiment (M=1) and examine the number of particles N and the noise ratio ϵ. We find that recovery is accurate and reliable for ϵ≤0.1, yielding correct identification of $K_{\text{QANR}}$ with less than 1% relative error in at least 98/100 trials. Increasing N from 500 to 8000 leads to minor improvements in accuracy for ϵ≤0.1, but otherwise has little effect, implying that for low to moderate noise levels the mean-field equations are readily identifiable even from smaller particle systems. For $\epsilon \;=\;10^{-1∕2}\approx 0.3162$ (see Fig. 4 (bottom right) for an example histogram), we observe a decrease in $\text{TPR}(\hat{w})$ (Fig. 5 middle panel) resulting from the generic identification of a linear diffusion term $v\partial_{xx}u$ with v≈0.05. Using that $\sqrt{2v}\approx \sqrt{2(0.05)}=\epsilon$, we can identify this as the best-fit *intrinsic* noise model. Furthermore, increases in N lead to reliable identification of the drift term, as measured by $\text{TPR}({\hat{w}}_{drift})$ (rightmost panel Fig. 5) which is the restriction of TPR to drift terms $L_{K}$ and $L_{V}$.

**Fig. 6.**
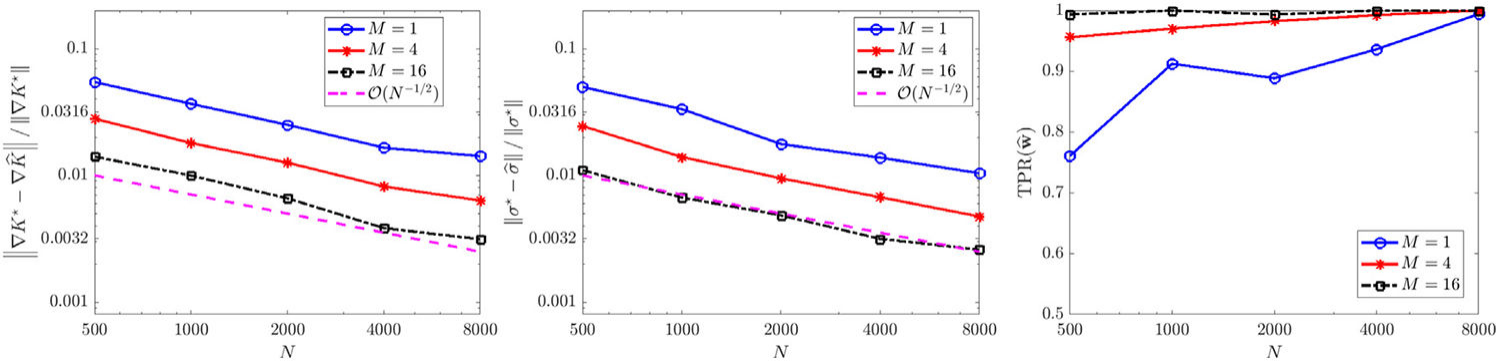
Recovery of (3.1) in one spatial dimension for $K^{⋆}=K_{\mathrm{QANR}}$ and $\sigma^{⋆}=\sqrt{2(0.1)}$.

**Fig. 7.**
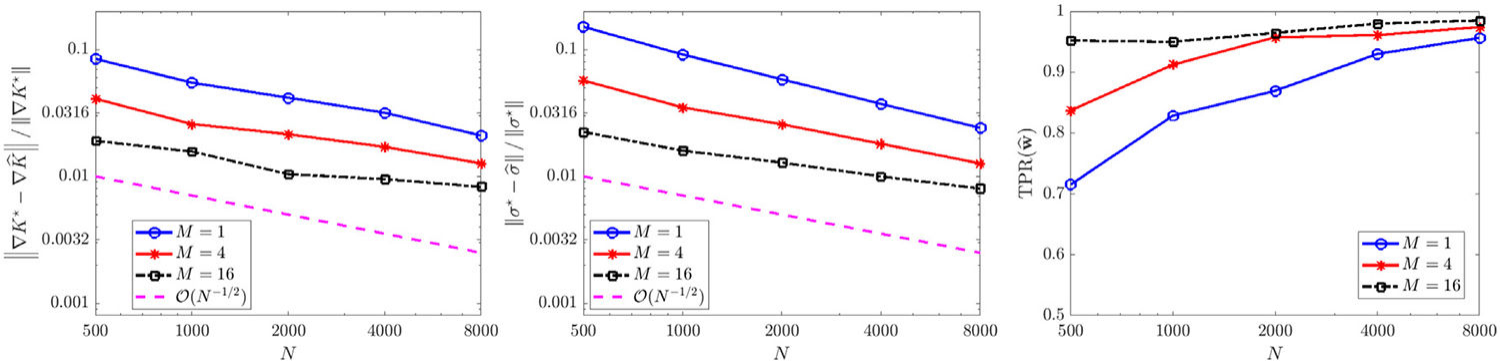
Recovery of (3.1) in one spatial dimension for $K^{⋆}=K_{\mathrm{QANR}}$ and $\sigma^{⋆}=\sqrt{2(0.1)}|x-2|$.

For constant diffusivity $\sigma (x)=\sqrt{2(0.1)}$ (Fig. 6), the full model is recovered with less than 3% errors in $\hat{K}$ and $\hat{\sigma }$ in at least 98/100 trials when the total particle count NM is at least 8000, and yields errors less than 1% for NM≥16,000. The error trends for $\hat{K}$ and $\hat{\sigma }$ in this case both strongly agree with the predicted $O(N^{-1∕2})$ rate. For non-constant diffusivity $\sigma (x)=\sqrt{2(0.1)}|x-2|$ (Fig. 7), we also observe robust recovery $\left(\text{TPR}(\hat{w})\geq 0.95\right)$ for NM≥8000 with error trends close to $O(N^{-1∕2})$, although the accuracy in $\hat{K}$ and $\hat{\sigma }$ is diminished due to the strong order $\Delta t^{1∕2}$ convergence of Euler–Maruyama applied to diffusivities σ that are unbounded in x [55].

### 5.3. Two-dimensional nonlocal model

We now discuss an example of singular interaction in two spatial dimensions using the logarithmic potential

(5.5) $$K(x)=\frac{1}{2\pi }\;\log|x|$$

with constant diffusivity $\sigma (x)\;=\;\sigma \;\in \{0,\frac{1}{\sqrt{4\pi }}\}$. This example corresponds to the parabolic–elliptic Keller–Segel model of chemotaxis, where $\sigma_{c}≔\frac{1}{\sqrt{4\pi }}$ is the critical diffusivity such that $\sigma >\sigma_{c}$ leads diffusion-dominated spreading of particles throughout the domain (vanishing particle density at every point in $R^{2}$) and $\sigma <\sigma_{c}$ leads to aggregation-dominated concentration of the particle density to the dirac-delta located at the center of mass of the initial particle density [44,56]. For σ=0 we examine the affect of additive i.i.d. measurement noise $\varepsilon ∼N(0,\epsilon^{2}\;\|X_{t}{\|}_{\text{RMS}}^{2})$ for ϵ∈ {0.01, 0.0316, 0.1, 0.316, 1}.

**Fig. 8.**
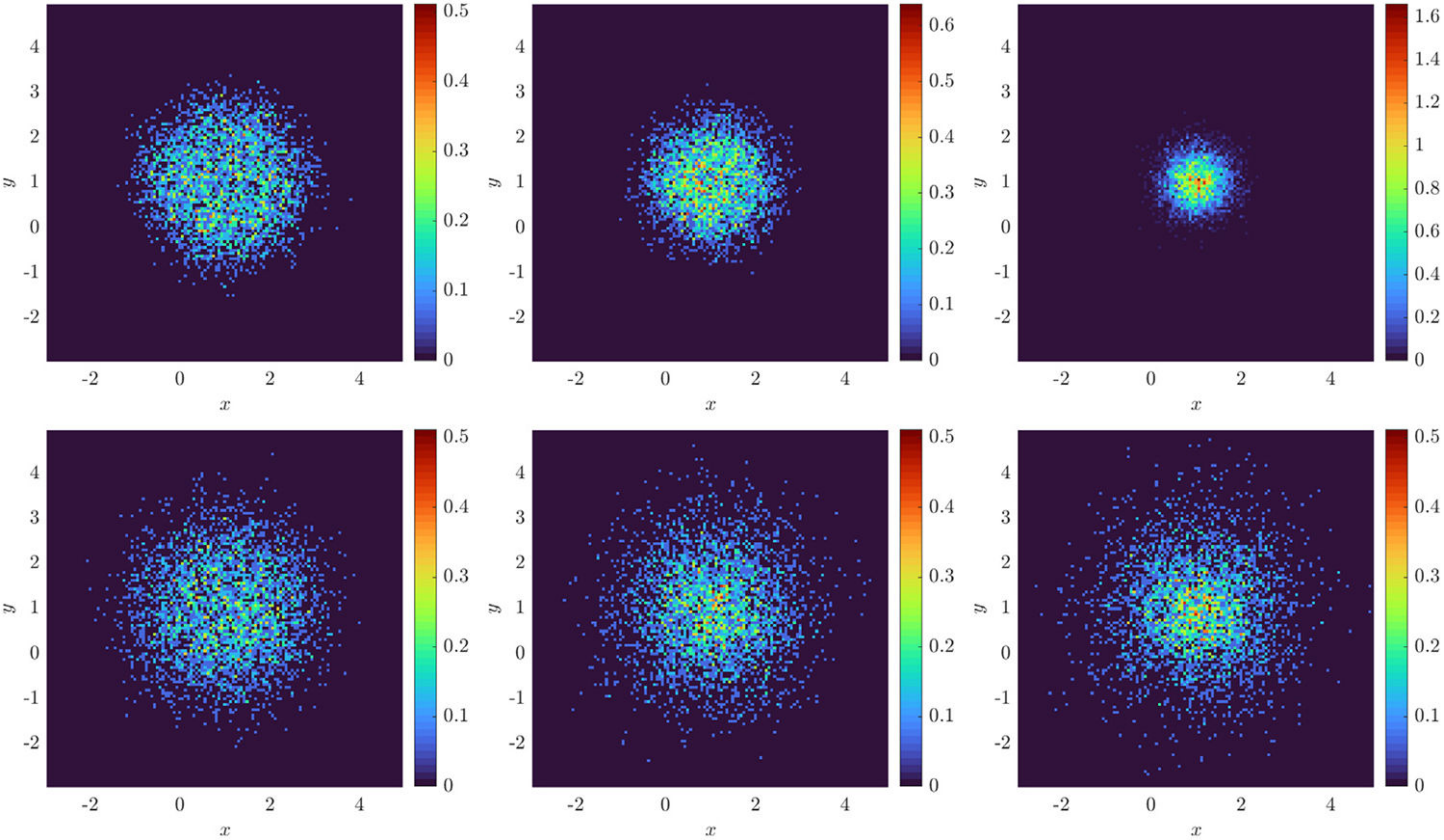
Histograms created from 4000 particles evolving under logarithmic attraction (Eq. (5.5) with varying noise levels at times (left to right) t=4, t=8, and t=12. Top: ϵ=0.316, σ=0 (extrinsic only). Bottom: ϵ=0, $\sigma ={\left(4\pi \right)}^{-1∕2}\approx 0.28$ (intrinsic only).

We simulate the particle system with a cutoff potential

(5.6) Kδ(x)={12π(log(δ)−1+∣x∣δ),∣x∣<δ12πlog∣x∣,∣x∣≥δ}

with δ=0.01, so that $K_{\delta }$ is Lipschitz and $\nabla K_{\delta }$ has a jump discontinuity at the origin. Initial particle positions are uniformly distributed on a disk of radius 2 and the particle position data consists of 81 timepoints recorded at a resolution Δt=0.1, coarsened from 0.0025. Histograms are created with 128 × 128 bins in x and y of sidelength h=0.0469 (see Fig. 8 for histogram snapshots over time). We examine $M=2^{0},\ldots ,2^{6}$ experiments with N=2000 or N=4000 particles.

**Fig. 9.**
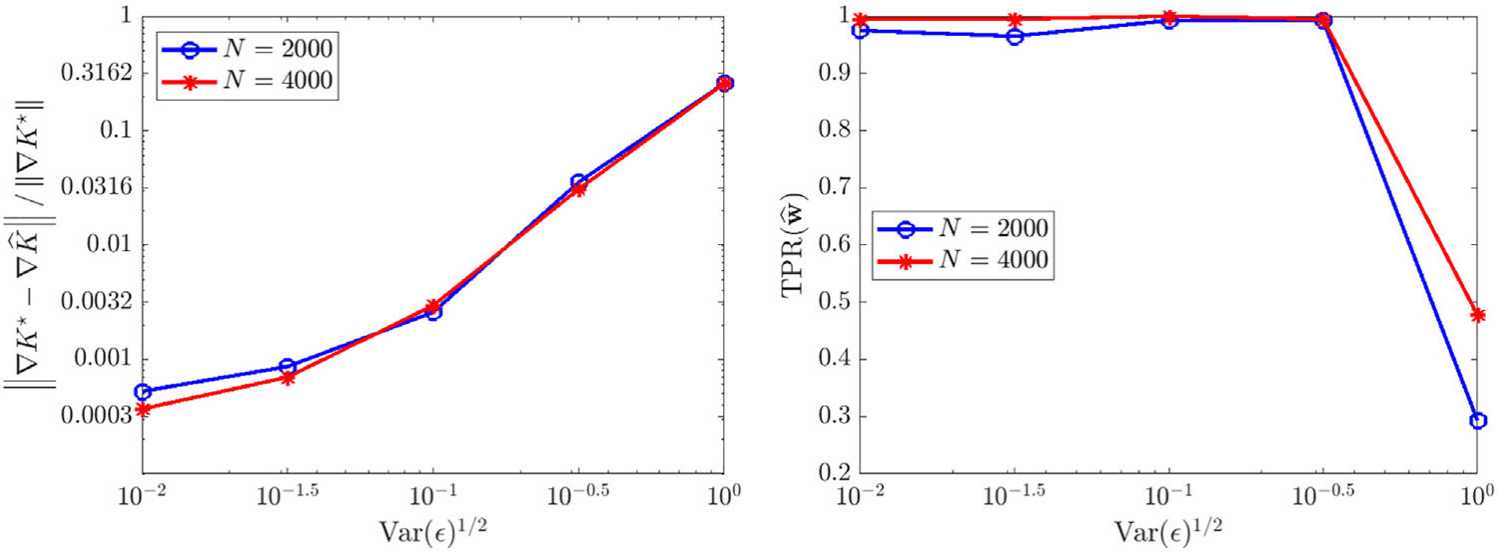
Recovery of (3.1) in two spatial dimensions with $K^{⋆}$ given by (5.5) from deterministic particles ($\sigma^{⋆}=0$) with extrinsic noise ϵ.

In Fig. 9 we observe a similar trend in the σ=0 case as in the 1D nonlocal example, namely that recovery for ϵ≤0.01 is robust with low errors in $\hat{K}$ (on the order of 0.0032), only in this case the full model is robustly recovered up to ϵ=0.316. At ϵ=1, with N=4000 the method frequently identifies a diffusion term vΔu with $v\approx 0.5=\epsilon^{2}∕2$, and for N=2000 the method occasionally identifies the backwards diffusion equation $\partial_{t}\mu_{t}\;=\;-\alpha \Delta \mu_{t}$, α>0. This is easily prevented by enforcing positivity of σ, however we leave this and other constraints as an extension for future work.

**Fig. 10.**
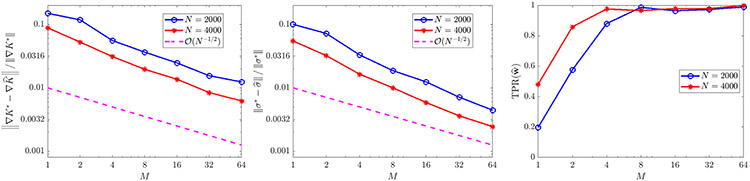
Recovery of (3.1) in two spatial dimensions with $K^{⋆}$ given by (5.5) and $\sigma^{⋆}=\frac{1}{\sqrt{4\pi }}$.

With diffusivity $\sigma \;=\;\frac{1}{\sqrt{4\pi }}$, we obtain $\text{TPR}(\hat{w})$ approximately greater than 0.95 for NM≥16,000 (Fig. 10, right), with an error trend in $\hat{K}$ following an $O(N^{-1∕2})$ rate, and a trend in $\hat{\sigma }$ of roughly $O(N^{-2∕3})$. Since convergence in M for any fixed N is not covered by the theorem above, this shows that combining multiple experiments may yield similar accuracy trends for moderately-sized particle systems.

## 6. Discussion

We have developed a weak-form method for sparse identification of governing equations for interacting particle systems using the formalism of mean-field equations. In particular, we have investigated two lines of inquiry, (1) is the mean-field setting applicable for inference from medium-size batches of particles? And (2) can a low-cost, low-regularity density approximation such as a histogram be used to enforce weak-form agreement with the mean-field PDE? We have demonstrated on several examples that the answer is yes to both questions, despite the fact that the mean-field equations are only valid in the limit of infinitely many particles (N→∞). This framework is suitable for systems of several thousand particles in one and two spatial dimensions, and we have proved convergence in N for the associated least-squares problem using simple histograms as approximate particle densities. In addition, the sparse regression approach allows one to identify the full system, including interaction potential K, local potential V, and diffusivity σ.

It was initially unclear whether the mean-field setting could be utilized in weak form for finite particle batches, hence this can be seen as a proof of concept for particle systems with N in the range 10^3^ – 10^5^. With convergence in N and low computational complexity, our weak-form approach is well-suited *as is* for much larger particle systems. In the opposite regime, for small fixed N, the authors of [26] show that their maximum likelihood-based method converges as M→∞ (i.e. in the limit of infinite experiments). While the same convergence does not hold for our weak-form method, the results in Section 5 suggest that in practice, combining M independent experiments each with N particles improves results. Furthermore, we include evidence in Appendix A.3 that even for small N, our method correctly identifies the mean-field model when M is large enough, with performance similar to that in [26]. We leave a full investigation of the interplay between M and N to future work.

In the operable regime of $N\;>\;10^{3}$, there is potential for improvements and extensions in many directions. On the subject of density estimation, histograms are highly efficient, yet they lead to piecewise-constant approximations of $\mu_{t}$ and hence O(h) errors. Choosing a density kernel G to achieve high-accuracy quadrature without sacrificing the O(N) runtime of histogram computation seems prudent, although one must be cautious about making assumptions on the smoothness of mean-field distribution $\mu_{t}$. For instance, in the 1D nonlocal example 5.2, discontinuities develop in $\mu_{t}$ for the case σ=0, hence a histogram approximation is more appropriate than using e.g. a Gaussian kernel.

The computational grid C, quadrature method $\langle \cdot ,\cdot \rangle_{h,\Delta t}$, and reference test function ψ may also be optimized further or adapted to specific problems. The approach chosen here of C equally-spaced and separable piecewise-polynomial ψ, along with integration using the trapezoidal quadrature, has several advantages, including high accuracy and fast computation using convolutions. However, this may need adjustment for higher dimensions. It might be advantageous to adapt C to the data Y, however this may prevent one from evaluating (G, b) using the FFT if a non-uniform grid results, hence increases the overall computational complexity. One could also use multiple reference test functions ψ. The possibilities of varying the test functions (within the smoothness requirements of the library L) have been largely unexplored in weak-form identification methods.

Several theoretical questions remain unanswered, namely model recovery statistics for finite N. As a consequence of Theorem 1, as well as convergence results on sequential thresholding [47], we have that G being full-rank and L containing the true model is sufficient to guarantee convergence $\hat{w}\to w^{⋆}$ as N→∞ at the rate $O(N^{-1∕2})$. Noise, whether extrinsic or intrinsic, for finite N may result in identification of an incorrect model when G is poorly-conditioned. The effect is more severe if the true model has a small coefficient, which requires a small threshold λ, which correspondingly may lead to a non-sparse solution. These are sensitivities of any sparse regression algorithm (see e.g. [57]) and accounting for the effect of noise and poor conditioning is an active area of research in equation discovery.

We also note that several researchers have focused on the uniqueness in kernel identifiability [34,58]. This issue does not directly apply to our scenario^21^ of identifying the triple (K, V, σ). Moreover, in the cases we considered, we do not see any identifiability issues (e.g. rank deficiency) even in the high noise case with low particle number. Quantifying the transition to identifiability as N→∞ as a function of the condition number κ(G) is an important subject for future work.

For extensions, the example system (5.2) and resulting homogenization motivates further study of effective equations for systems with complex microstructure. In other fields this is described as *coarse-graining*. A related line of study is inference of 2nd-order particle systems, as explored in [32], which often lead to an infinite hierarchy of mean-field equations. Our weak-form approach may provide a principled method for truncated and closing such hierarchies using particle data. Another extension is to enforce convex constraints in the regression problem, such as lower bounds on diffusivity, or K with long-range attraction depending on the distribution $\rho_{\pi }\;\in \;P([0,\infty ))$ of pairwise distances (see [26] for further use of $\rho_{\pi }$). Finally, the framework we have introduced can easily be used to find nonlocal models from continuous solution data (e.g. given U instead of Y), whereby questions of nonlocal representations of models can be investigated.

Lastly, we note that MATLAB code is available at https://github.com/MathBioCU/WSINDy_IPS.

## Figures and Tables

**Table 1:** Notations used throughout.

Variable	Definition	Domain
K	Pairwise interaction potential	$L_{\mathrm{loc}}^{1}(R^{d},R)$
V	Local potential	$C(R^{d},R)$
σ	Diffusivity	$C(R^{d},R^{d\times d})$
N	Number of particles per experiment	{2, 3,…}
d	Dimension of latent space	N
T	Final time	(0, ∞)
(Ω,B, P, ${\left(F\right)}_{t\geq 0}$, ${\left(B_{t}^{\left(i\right)}\right)}_{i=1}^{N}$)	Filtered probability space	
$R^{d}$	Independent Ω,B Brownian motions on (P, ${\left(F\right)}_{t\geq 0}$, ${\left(B_{t}^{\left(i\right)}\right)}_{i=1}^{N}$, $X_{t}^{\left(i\right)}$)	
ith	t particle in the particle system (1.1) at time $R^{d}$	$X_{t}$
N	t-particle system (1.1) at time $R^{Nd}$	$\mu_{t}^{N}$
	Empirical measure of $X_{t}$	$P(R^{d})$
$F_{t}^{N}$	Distribution of $X_{t}$	$P(R^{Nd})$
$X_{t}$	Mean-field process (3.2) at time t	$R^{d}$
$\mu_{t}$	Distribution of $X_{t}$	$P(R^{d})$
t	L discrete timepoints	[0, [0,T]]
$X_{t}$	Collection of M independent samples of $X_{t}$ at t	$R^{MLNd}$
$Y_{t}$	Sample of $X_{t}$ corrupted with i.i.d. additive noise	$R^{MLNd}$
$U_{t}$	Approximate density from particle positions	$P(R^{d})$
G	Density kernel mapping $\mu_{t}^{N}$ to $U_{t}$	$L^{1}(R^{d}\times R^{d},R)$
D	Spatial support of $U_{t}$, t∈[0,T]	Compact subset of $R^{d}$
C	Discretization of D	
$U_{t}$	Discrete approximate density $U_{t}(C)$	
${\langle \cdot ,\cdot \rangle }_{h}$	semi-discrete inner product, trapezoidal rule over C	
${\langle \cdot ,\cdot \rangle }_{h,\Delta t}$	Fully-discrete inner product, trapezoidal rule over C×t	
$L_{K}$	Library of candidate interaction forces	
$L_{V}$	Library of candidate local forces	
$L_{\sigma }$	Library of candidate diffusivities	
L	($L_{K}$, $L_{V}$, $L_{\sigma }$)	
Ψ	Set of n test functions ${\left(\psi_{k}\right)}_{k=1}^{n}$	$C^{2}(R^{d}\times (0,T))$
$\phi_{m,p}(v;\Delta )$	Test functions used in this work (Eq. (4.4)	
λ	Set of sparsity thresholds	
L	Loss function for sparsity thresholds (Eq. (4.6)	

**Table 2:** Trial function library for local 2D example (Section 5.1).

Mean-field term	Trial function library
∇⋅(U∇K∗U)	$\nabla \cdot (U\nabla {|x|}^{m}*U)$, m∈{1,2,3,4,5,6,7} {1, 2, 3, 4, 5, 6, 7}
∇⋅(U∇V)	$\partial_{x_{i}}(U\;\cos(mx_{1})\cos(nx_{2}))$, (m,n)∈{0,1,2,3,4,5} {0, 1, 2, 3, 4, 5}, i∈{1,2} {1, 2}
$\frac{1}{2}\sum_{i,j=1}^{d}\frac{\partial^{2}{\left(U\sigma \sigma^{T}\right)}_{ij}}{\partial x_{i}\partial x_{j}}$	$\Delta (U\;\cos(mx_{1})\cos(nx_{2}))$, (m,n)∈{0,1,2,3,4,5} {0, 1, 2, 3, 4, 5}

**Table 3:** Trial function library for nonlocal 1D example (Section 5.2).

Mean-field term	Trial function library
∇⋅(U∇K∗U)	$\nabla \cdot (U\nabla {|x|}^{m}*U)$, m∈{1,2,3,4,5,6,7} {1, 2, 3, 4, 5, 6, 7}
∇⋅(U∇V)	$\partial_{x}(Ux^{m})$, m∈{0,2,3,4,5,6,7,8} {0, 2, 3, 4, 5, 6, 7, 8}
$\frac{1}{2}\sum_{i,j=1}^{d}\frac{\partial^{2}{\left(U\sigma \sigma^{T}\right)}_{ij}}{\partial x_{i}\partial x_{j}}$	$\partial_{xx}(Ux^{m})$, m∈{0,1,2,3,4,5,6,7,8} {0, 1, 2, 3, 4, 5, 6, 7, 8}

**Table 4:** Trial function library for nonlocal 2D example (Section 5.3). Interaction potentials ${\left[K\right]}_{\delta }$ indicate cutoff potentials of the form (5.6) with δ=0.01 such that the resulting potential is Lipschitz.

Mean-field term	Trial function library
∇⋅(U∇K∗U)	{∇⋅(U∇∣x∣m∗U)m∈{2,3,4,5,6}∇⋅(U∇[∣x∣1∕2]δ∗U)∇⋅(U∇[∣x∣(log∣x∣−1)]δ∗U)∇⋅(U∇[log∣x∣]δ∗U)}
∇⋅(U∇V)	$\partial_{x_{i}}\left(Ux_{1}^{m}x_{2}^{n}\right)\;0\leq m+n\leq 5,i\in \{1,2\}$
$\frac{1}{2}\sum_{i,j=1}^{d}\frac{\partial^{2}{\left(U\sigma \sigma^{T}\right)}_{ij}}{\partial x_{i}\partial x_{j}}$	$\Delta (U\;\cos(mx_{1})\cos(nx_{2}))$, (m,n)∈{0,1,2}

**FIG. 11.**
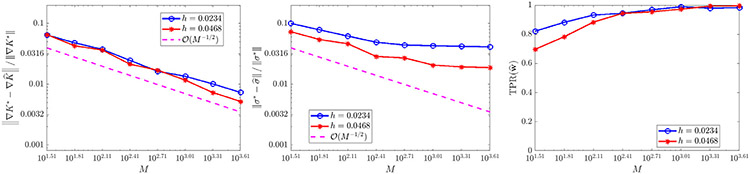
Recovery of (3.1) in one spatial dimension for $C_{p}$ and T with only ψ particles per experiment.

## Footnotes

1 We define the pth moment of a probability measure μ for p≥0 by $M_{p}(\mu )≔\int_{R^{d}}{|x|}^{p}d\mu (x)$.

2 The set D−D is defined D−D={x−y:(x,y)∈D×D}.

3 By $I_{d}$ we mean the identity in $R^{d}$.

4 We use the notation $t\to \mu_{t}$ to denote the evolution of probability measures. Subscripts will not be used to denote differentiation.

5 Meaning that for all continuous bounded functions $\phi :R^{d}\to R$, $\int_{R^{d}}d\mu_{t}^{N}(x)\to \int_{R^{d}}d\mu_{t}(x)$.

6 For a function $f:R^{d}\to Y$, where Y is a metric space with metric ρ, we define Lip(f) by

$$\text{Lip}(f)≔\underset{x,y\in R^{d}}{\text{sup}}\frac{\rho (f(x),f(y))}{|x-y|}$$

where ∣⋅∣ denotes the Euclidean norm. We say f is Lipschitz when Lip(f)<∞. Also, ${\left\|f\right\|}_{C^{1}}≔{\left\|f\right\|}_{\infty }+\sum_{i=1}^{d}{\left\|\frac{\partial f}{\partial x_{i}}\right\|}_{\infty }$.

7 The indicator function is defined 1A(x)≔{1,x∈A0,x∉A⋅}.

8 In this case (4.2) is the variance of a Monte-Carlo estimator for ∫ψ(x)dμ(x).

9 Details of the libraries used in examples can be found in Tables 2-4 in Appendix A.1.

10 This is not true in domains with boundaries, where nonlocalities can be seen to impart mean translation [42].

11 Note that this is feasible because the STLS algorithm terminates in finitely many iterations.

12 The Moore-Penrose inverse $A^{†}$ is defined for a rank-r matrix A using the reduced SVD $A=U_{r}\Sigma_{r}V_{r}^{*}$ as $A^{†}≔V_{r}\Sigma_{r}^{-1}U_{r}^{*}$. The subscript r denotes restriction to the first r columns.

13 In particular, correlations result in large mutual incoherence, which renders algorithms such as Basis Pursuit, Orthogonal Matching Pursuit, and Hard Thresholding Pursuit useless (see [48, Chapter 5] for details).

14 Note that C−C is simply C shifted to lie in the positive orthant $\left\{x\in R^{d}:x_{\ell }\geq 0,\;1\leq \ell \leq d\right\}$ and reflected through each coordinate plane $x_{\ell }=0$. In this way C−C discretizes the set $D-D≔\{x-y\in R^{d}:(x,y)\in D\times D\}$ containing all observed interparticle distances.

15 We neglect the cost of computing the histogram U and evaluating ψ(C), together amounting to an additional O(NML+∣C∣) flops, as these terms are lower order and reused in each column of G and b.

16 For $A\in R^{d\times d}$ the Frobenius norm is defined ${\left\|A\right\|}_{F}=\sqrt{\mathrm{Tr}(A^{T}A)}$

17 ${\left\|G^{†}\right\|}_{q}$ is the induced matrix q-norm of $G^{†}$.

18 Note that the opposite convergence holds for the algorithm introduced in [26]: N-fixed, M→∞ results in recovery of K.

19 For example, identification of the true model $\left(\mathrm{supp}(\hat{w})=\mathrm{supp}(w^{⋆})\right)$ results in a $\mathrm{TPR}(\hat{w})=1$, while identification of only half of the correct nonzero terms and no additional falsely identified terms results in $\mathrm{TPR}(\hat{w})=0.5$.

20 Since the model is local, (5.2) is the Fokker-Planck equation for the distribution of each particle, rather than only in the limit of infinite particles.

21 E.g. due to multiple representations of the drift combining both nonlocal and local terms – see Section 4.1.4

## Appendix

### A.1. Specifications for examples

In Tables 2-5 we include hyperparameter specifications and resulting attributes of Algorithm 4.1 applied to the three examples in Section 5. In particular, we report the typical walltime in Table 5, showing that on each example Algorithm 4.1 learns the mean-field equation from a dataset with ~64,000 particles in under 10 s.

**Table 5:** Discretization parameters and general information for examples. The number of nonzeros in the true weight vector $\|w^{⋆}{\|}_{0}$ is given for each parameter set examined. Namely, for the local 2D example, ω=1 results in a 4-term model, while the homogenized case ω=20 results in a three-term model. For the nonlocal 1D example, $\sigma \;\in \;\{0,\sqrt{2(0.1)},\sqrt{2(0.1)}|x-2|\}$ result in 2-term, 3-term, and 5-term models, respectively, and for the nonlocal 2D example $\sigma \in \{0,(4\pi {)}^{-1}\}$ results in 1-term and 2-term models. The norm ${\left\|G^{†}\right\|}_{1}$, condition number $\kappa_{2}(G)$ and walltime are listed for representative samples with 64,000 total particles.

Example	($m_{x}$, $m_{t}$)	($p_{x}$, $p_{t}$)	($s_{x}$, $s_{t}$)	size(U)	(h, Δt)
Local 2D	(31,16)	(5,3)	(10,5)	128 × 128 × 101	(0.078, 0.02)
Nonlocal 1D	(29,8)	(5,3)	(5,1)	256 × 101	(0.023, 0.01)
Nonlocal 2D	(25,8)	(5,3)	(8,1)	128 × 128 × 81	(0.047, 0.1)
Example	$\|w^{⋆}{\|}_{0}$	size(G)	$\|G^{†}{\|}_{1}$	$\kappa_{2}(G)$	Walltime
Local 2D	{4, 3}	686 × 85	2.0 × 10^3^	3.0 × 10^7^	9.2 s
Nonlocal 1D	{2, 3, 5}	3400 × 24	1.3 × 10^5^	8.7 × 10^8^	0.7 s
Nonlocal 2D	{1, 2}	6500 × 59	1.1 × 10^4^	6.4 × 10^6^	8.5 s

### A.2. Derivation of homogenized equation (5.3)

We briefly provide a derivation of the homogenized equation (5.3) in the static case. Let $\Omega \in R^{d}$ be an open bounded domain with smooth boundary and $T^{d}$ be the d-dimensional torus. Let $a(x,y)\;:\Omega \times T^{d}\to R$ be continuous and uniformly bounded below,

$$a(x,y)\geq \alpha >0,\;\;(x,y)\in \Omega \times T^{d}.$$

Then for any $f\in L^{2}(\Omega )$, the equation

$$-\Delta \;(a(x,x∕\epsilon )u^{\epsilon }(x))=f(x),\;\;u^{\epsilon }{|}_{\partial \Omega }=0$$

has a unique weak solution $u^{\epsilon }\in L^{2}(\Omega )$ given by

$$u^{\epsilon }(x)=\frac{\left(Gf)(x\right)}{a(x,x∕\epsilon )},$$

where G is the Green’s function for $(-\Delta {)}^{-1}$ with homogeneous Dirichlet boundary conditions on ∂Ω. By the coercivity of a we have that $\|u^{\epsilon }{\|}_{L^{2}(\Omega )}$ is uniformly bounded in ϵ. By the lemma in [59, Section 2.4], up to a subsequence $\{\epsilon_{j}{\}}_{j\in N}$, there exists a function u(x,y) periodic in its second variable such that for any continuous function ϕ(x,x∕ϵ), we have

$$\underset{\epsilon \to 0}{\text{lim}}\int u^{\epsilon }(x)\phi (x,x∕\epsilon )dx=\iint u(x,y)\phi (x,y)dydx.$$

Setting ϕ(x,y)=ϕ(x), we see that on the same subsequence, $u^{\epsilon }⇀\int u(x,y)dy$. Applying the same lemma to the constant series $u^{\epsilon }=1$ and letting $\phi (x,x∕\epsilon )=\phi (x)a^{-1}(x,x∕\epsilon )$, we see that (up to possibly a second subsequence),

$$a^{-1}(x,x∕\epsilon )⇀\int \frac{dy}{a(x,y)}.$$

Letting $a^{*}(x)\;≔{\left(\int \frac{dy}{a(x,y)}\right)}^{-1}$ and putting together the previous limits, we see that

$$u^{\epsilon }(x)⇀u^{*}(x)≔\int u(x,y)dy=(Gf)(x)\int \frac{dy}{a(x,y)}≕\frac{\left(Gf)(x\right)}{a^{*}(x)},$$

and hence $u^{*}$ solves the homogenized equation

$$\Delta \;(a^{*}u^{*})=f.$$

### A.3. Recovery for small N and large M

The related maximum-likelihood approach [26] is shown to be suitable for small N and large M, hence a natural line of inquiry is the performance of Algorithm 4.1 in this regime. Theorem 1 does not apply to this regime, and in fact convergence of the algorithm is not expected: letting $U_{t}^{M,N}=\frac{1}{M}\sum_{m=1}^{M}U_{t}^{(m),N}$ where $U_{t}^{(m),N}$ is the approximate density constructed from experiment m with N particles, we have the weak-measure convergence $U_{t}^{M,N}\to \rho_{t}^{(1),N}$ as M→∞, where $\rho_{t}^{(1),N}$ is the 1-particle marginal of the distribution of $X_{t}$ in $R^{Nd}$. Unlike the mean-field distribution $\mu_{t}$, $\rho_{t}^{(1),N}$ is not a weak solution to the mean-field Fokker–Planck equation (3.1), instead we have

$$\partial_{t}\;\rho_{t}^{(1),N}=\frac{N-1}{N}\nabla \cdot \int_{R^{d}}\nabla K(x-y)\rho_{t}^{(2),N}(x,y)dy+\nabla \cdot \left(\nabla V\rho_{t}^{(1),N}\right)\;\;+\frac{1}{2}\sum_{i,j=1}^{d}\partial_{x_{i}x_{j}}(\sigma \sigma^{T}\;\rho_{t}^{(1),N}),$$

holding weakly, which depends on the 2-particle marginal $\rho_{t}^{(2),N}$ [35]. Nevertheless, using the 1D nonlocal example in Section 5.2 with $\sigma =\sqrt{2(0.1)}\approx 0.45$, we observe in Fig. 11 (right panel) that our weak-form algorithm correctly identifies the model in > 96% of trials with just N=10 particles per experiment when $M\;\in \;[2^{10},2^{12}]$, and that error in K (left panel) follows a $O(M^{-1∕2})$ trend. At $M=4096\approx 10^{3.61}$ experiments the error^22^ in K is less than 1% and the runtime is approximately 0.9 s. The lack of convergence in M is reflected in the diffusivity (middle panel of Fig. 11), where the error appears to plateau at around 1.7% for h≈0.0468 and at 3.5% for h≈0.0234. The lower resolution (larger binwidth h) appears to yield slightly better results, possibly indicating that larger h produces a coarse-graining effect such that $\rho^{(2),N}\approx \rho^{(1),N}⊗\rho^{(1),N}$ over larger distances, although this effect deserves more thorough study in future work.

### A.4. Technical lemmas

We now prove Lemmas 2-4 under Assumption H. First, some consequences of Assumption H. (I) The η-Hölder continuity of sample paths (H.1) implies that for each t∈[0,T],

$$\int_{R^{d}}|x{|}^{p}d\mu_{t}^{N}=\frac{1}{N}\sum_{i=1}^{N}|X_{t}^{\left(i\right)}{|}^{p}\leq \frac{2^{p}}{N}\sum_{i=1}^{N}|X_{0}^{\left(i\right)}{|}^{p}+C_{\eta }2^{p}t^{p\eta }.$$

Together with the pth moment bound on $\mu_{0}$ (H.2), this implies

(A.1)
$$E\left[\underset{t\leq T}{\text{sup}}\int_{R^{d}}|x{|}^{p}d\mu_{t}^{N}\right]\leq 2^{p}(M_{p}+C_{\eta }T^{p\eta }),$$

independent of N. (II) The growth bounds on $\nabla K^{⋆}$, $\nabla V^{⋆}$, and $\sigma^{⋆}$ (H.3)-(H.4) imply that for some C>0,

(A.2)
$$|\nabla K^{⋆}(x)|+|\nabla V^{*}(x)|+\|\sigma^{⋆}(x)(\sigma^{⋆}(x){)}^{T}{\|}_{F}\leq C(1+|x{|}^{p}),$$

where ${\left\|\cdot \right\|}_{F}$ is the Frobenius norm.

**Proof of** Lemma 2. Applying Itô’s formula to the process $\frac{1}{N}\sum_{i=1}^{N}\psi (X^{\left(i\right)},t)$, we get that

$$L(\mu^{N},\psi ,\langle \cdot ,\cdot \rangle )=\frac{1}{N}\sum_{i=1}^{N}\int_{0}^{T}\nabla \psi (X_{t}^{\left(i\right)},t{)}^{T}\sigma^{⋆}(X_{t}^{\left(i\right)})dB_{t}^{\left(i\right)}.$$

Note that each integral on the right-hand side is a local martingale, since (A.2) and (H.5) ensure boundedness of $\nabla \psi {\left(x,t\right)}^{T}\sigma^{⋆}(x)$ over any compact set in $R^{d}$, hence has mean zero. By independence of the Brownian motions $B_{t}^{\left(i\right)}$, exchangeability of $X_{t}^{\left(i\right)}$, the moment bound (A.1), and the growth bounds on σ (H.4), the Itô isometry gives us

$$E\;[L(\mu^{N},\psi ,\langle \cdot ,\cdot ,\rangle {)}^{2}]\;\;=\frac{1}{N}\int_{0}^{T}E_{X∼\rho_{t}^{\left(1\right)}}\left[|\nabla \psi (X,t{)}^{T}\sigma^{⋆}(X){|}^{2}\right]dt\;\;=\frac{1}{N}\int_{0}^{T}E\left[\int_{R^{d}}|\nabla \psi (x,t{)}^{T}\sigma^{⋆}(x){|}^{2}\;d\mu_{t}^{N}(x)\right]dt\;\;\leq \frac{C^{\prime }}{N}\|\nabla \psi {\|}_{2,\infty }^{2}\int_{0}^{T}E\left[1+\int_{R^{d}}|x{|}^{p}d\mu_{t}^{N}(x)\right]dt\;\;\leq CN^{-1}$$

where $K^{⋆}=K_{\mathrm{QANR}}$ depends on $\sigma^{⋆}=\sqrt{2(0.1)}$, N=10, C, and $M_{p}$. The result follows from Jensen’s inequality.^23^

**Proof of** Lemma 3. Using the notation $f^{C}$ from Lemma 1 to denote piecewise constant approximation of a function f over the domain D using the grid C, we have

$$L(U,\psi ,\langle \cdot ,\cdot \rangle_{h})-L(\mu^{N},\psi ,\langle \cdot ,\cdot \rangle )\;\;=-\underset{E_{\text{interact}}}{\underset{︸}{\left(\langle (\nabla \psi \cdot ((\nabla K^{⋆}{)}^{C}*\mu^{N}){)}^{C},\mu^{N}\rangle -\langle \nabla \psi \cdot \nabla K^{⋆}*\mu^{N},\mu^{N}\rangle \right)}}\;\;+\langle \partial_{t}\psi^{C}-\partial_{t}\psi ,\mu^{N}\rangle -\langle (\nabla \psi \cdot \nabla V^{*}{)}^{C}-\nabla \psi \cdot \nabla V^{*},\mu^{N}\rangle \;\;+\frac{1}{2}\langle \text{Tr}(\nabla^{2}\psi \sigma^{⋆}(\sigma^{⋆}{)}^{T}{)}^{C}-\text{Tr}(\nabla^{2}\psi \sigma^{⋆}(\sigma^{⋆}{)}^{T}),\mu^{N}\rangle \;\;=E_{\text{interact}}+E_{\text{linear}}.$$

The right-hand side includes an interaction error $E_{\text{interact}}$ followed by a sum $E_{\text{linear}}$ of terms that are linear in the difference between a locally Lipschitz function and its piecewise constant approximation. Hence, we can bound $E_{\text{linear}}$ using smoothness of ψ (H.5), the moment assumptions on $\mu_{t}^{N}$ (H.2), and the growth assumptions on V and σ (H.3)-(H.4). Specifically, for $x\in B_{k}$ with center $c_{k}$, the growth assumptions imply

$$|\nabla \psi (x)\;\cdot \;\nabla V^{⋆}(x)-\nabla \psi (c_{k})\;\cdot \;\nabla V^{⋆}(c_{k})|\;\leq Ch\left(\left(\|\nabla \psi {\|}_{2,\infty }+\text{Lip}(\nabla \psi ))(1+|x{|}^{p}\right)\right)\;|\text{Tr}\;(\nabla^{2}\psi (x)\sigma^{⋆}(x)(\sigma^{⋆}(x){)}^{T})-\text{Tr}\;(\nabla^{2}\psi (c_{k})\sigma^{⋆}(c_{k})(\sigma^{⋆}(c_{k}){)}^{T})\;|\;\;\leq C^{\prime }h\left(\left(\|\nabla^{2}\psi {\|}_{F,\infty }+\text{Lip}(\nabla^{2}\psi ))(1+|x{|}^{p}\right)\right)$$

for C and $C^{\prime }$ depending on p, d, and $C_{p}$, hence

(A.3)
$$|E_{\text{linear}}|\leq C^{\prime \prime }\;\underset{|\alpha |\leq 2}{\text{sup}}\;\text{Lip}(\partial^{\alpha }\psi )\left(T+\int_{0}^{T}\int_{R^{d}}|x{|}^{p}d\mu_{t}^{N}dt\right)h.$$

Similarly, for the interaction error we use that for $x\in B_{k}$ and $y\in B_{j}$ with centers $c_{k}$ and $c_{j}$, we have

$$|\nabla \psi (c_{k})\cdot \nabla K^{⋆}(c_{k}-c_{j})-\nabla \psi (x)\cdot \nabla K^{⋆}(x-y)|\;\;\leq |\nabla \psi (c_{k})||\nabla K^{⋆}(c_{k}-c_{j})-\nabla K^{⋆}(x-y)|\;\;+|\nabla \psi (c_{k})-\nabla \psi (x)||\nabla K^{⋆}(x-y)|\;\;\leq C^{\prime \prime \prime }h\left(\left\|\nabla \psi {\|}_{2,\infty }+\text{Lip}(\nabla \psi \right)\right)(1+|x-y{|}^{p})$$

with $C^{\prime \prime \prime }$ also depending on p, d, and $C_{p}$. From this we have

(A.4)
$$|E_{\text{interact}}|\leq C^{\prime \prime \prime \prime }\left(T+\int_{0}^{T}\int_{R^{d}}\int_{R^{d}}|x-y{|}^{p}d\mu_{t}^{N}(y)d\mu_{t}^{N}(x)dt\right)h.$$

The result follows from taking expectation and using the moment bound (A.1), where the final constant C depends on p, d, $C_{p}$, $M_{p}$, T, η, and ψ.

**Proof of** Lemma 4. Again rewriting the spatial trapezoidal-rule integration in the form $\int_{R^{d}}\varphi^{C}(x)d\mu_{t}^{N}$, we see that

(A.5)
$$L(U,\psi ,\langle \cdot ,\cdot \rangle_{h})-L(U,\psi ,\langle \cdot ,\cdot \rangle_{h,\Delta t})$$

reduces to four terms of the form

A(φ)≔1N∑i=1N(∫0TφC(Xt(i))dt)(−Δt2∑ℓ=1L(φC(Xtℓ+1(i))+φC(Xtℓ(i))),

for $\varphi \in \left\{\partial_{t}\psi ,\nabla \psi \cdot \nabla V^{⋆},\mathrm{Tr}(\nabla^{2}\psi \sigma^{⋆}{\left(\sigma^{⋆}\right)}^{T}),\nabla \psi \cdot \nabla K^{⋆}*\mu_{t}^{N}\right\}$. Similarly to the bounds derived for $|\varphi (x)-\varphi^{C}(x)|$ in Lemma 3, the growth bounds on $K^{⋆}$, $V^{⋆}$ and $\sigma^{⋆}$ imply in general that

$$|\varphi (x)-\varphi (y)|\leq C|x-y|\;(1+\max\{|x|,|y|{\}}^{p}).$$

Rewriting the summands in A(φ),

$$\int_{0}^{T}\varphi^{C}(X_{t}^{\left(i\right)})dt-\frac{\Delta t}{2}\sum_{\ell =1}^{L}\left(\varphi^{C}(X_{t_{\ell +1}}^{\left(i\right)})+\varphi^{C}(X_{t_{\ell }}^{\left(i\right)})\right)\;=\sum_{\ell =1}^{L}\underset{I_{1}}{\underset{︸}{\int_{t_{\ell }}^{t_{\ell +1}}\left(\frac{t-t_{\ell }}{\Delta t}\right)(\varphi^{C}(X_{t}^{\left(i\right)})-\varphi^{C}(X_{t_{\ell +1}}^{\left(i\right)}))dt}}\;\;+\underset{I_{2}}{\underset{︸}{\int_{t_{\ell }}^{t_{\ell +1}}\left(\frac{t_{\ell +1}-t}{\Delta t}\right)(\varphi^{C}(X_{t}^{\left(i\right)})-\varphi^{C}(X_{t_{\ell }}^{\left(i\right)}))dt,}}$$

and using

$$|\varphi^{C}(x)-\varphi^{C}(y)|\leq |\varphi (x)-\varphi (c_{k})|+|\varphi (x)-\varphi (y)|+|\varphi (y)-\varphi (c_{\ell })|\;\;\leq C(2h+|x-y|)(1+\max\{|x|,|y|{\}}^{p})$$

where $x\in B_{k}$ and $y\in B_{\ell }$, we see that for $I_{1}$,

$$|\int_{t_{\ell }}^{t_{\ell +1}}\left(\frac{t-t_{\ell }}{\Delta t}\right)(\varphi^{C}(X_{t}^{\left(i\right)})-\varphi^{C}(X_{t_{\ell +1}}^{\left(i\right)}))dt|\;\leq \int_{t_{\ell }}^{t_{\ell +1}}\left(\frac{t-t_{\ell }}{\Delta t}\right)C(2h+|X_{t}^{\left(i\right)}-X_{t_{\ell +1}}^{\left(i\right)}|)(1+\max\{|X_{t}^{\left(i\right)}|,|X_{t_{\ell +1}}^{\left(i\right)}|{\}}^{p})dt\;\leq \int_{t_{\ell }}^{t_{\ell +1}}\left(\frac{t-t_{\ell }}{\Delta t}\right)C^{\prime }(2h+|t_{\ell +1}-t{|}^{\eta }|)(1+\max\{|X_{t}^{\left(i\right)}|,|X_{t_{\ell +1}}^{\left(i\right)}|{\}}^{p})dt.$$

Taking expectation on both sides and using the moment bound (A.1), we get

$$E\left[|\int_{t_{\ell }}^{t_{\ell +1}}\left(\frac{t-t_{\ell }}{\Delta t}\right)(\varphi^{C}(X_{t}^{\left(i\right)})-\varphi^{C}(X_{t_{\ell +1}}^{\left(i\right)}))dt|\right]\;\leq C\;(\Delta th+\Delta t^{1+\eta }).$$

We get the same bound for $I_{2}$. Summing over ℓ, and taking the average in i, we then get

$$E\;[|A(\varphi )|]\leq C(h+\Delta t^{\eta }),$$

which implies the desired bound on the difference (A.5).

22 For comparison, in [26] Fig. 4 the error in recovering K using the maximum-likelihood approach on an opinion dynamics example for $M=10^{3.6}$, N=10, and σ=0.5 is approximately 100 × 10^−1.2^% = 6.3%. 23 ${\left\|f\right\|}_{p,q}$ for vector-valued functions $f:R^{d}\to R^{d}$ denotes the $L^{q}$ norm over x of the $\ell^{p}$ norm of f(x). Also recall that $\rho_{t}^{\left(1\right)}$ is the $X_{t}^{\left(1\right)}$-marginal of the process $X_{t}\in R^{dN}$.
